# Associations between attainment of incentivised primary care indicators and incident diabetic retinopathy in England: a population-based historical cohort study

**DOI:** 10.1186/s12916-021-01966-x

**Published:** 2021-04-16

**Authors:** Ailsa J. McKay, Laura H. Gunn, Thirunavukkarasu Sathish, Eszter Vamos, Manjula Nugawela, Azeem Majeed, German Molina, Sobha Sivaprasad

**Affiliations:** 1 Department of Primary Care and Public Health, Imperial College London, London, UK; 2 Department of Public Health Sciences, University of North Carolina (UNC) at Charlotte, Charlotte, NC 28223 USA; 3 School of Data Science, UNC Charlotte, Charlotte, USA; 4 Centre for Population Health Sciences, Lee Kong Chian School of Medicine, Nanyang Technological University, Singapore, 308232 Singapore; 5 Population Health Research Institute (PHRI), McMaster University, Hamilton, ON L8L 2X2 Canada; 6 Institute of Ophthalmology, UCL and NIHR Moorfields Biomedical Retinal Research, UCL and Moorfields Eye Hospital, 162, City Road, London, EV1V 2PD UK

**Keywords:** Type 2 Diabetes, Retinopathy, General Practice, Glycated haemoglobin, Blood pressure, Cholesterol

## Abstract

**Background:**

The associations between England’s incentivised primary care-based diabetes prevention activities and hard clinical endpoints remain unclear. We aimed to examine the associations between attainment of primary care indicators and incident diabetic retinopathy (DR) among people with type 2 diabetes.

**Methods:**

A historical cohort (*n* = 60,094) of people aged ≥ 18 years with type 2 diabetes and no DR at baseline was obtained from the UK Clinical Practice Research Datalink (CPRD). Exposures included attainment of the Quality and Outcomes Framework (QOF) HbA1c (≤ 7.5% or 59 mmol/mol), blood pressure (≤ 140/80 mmHg), and cholesterol (≤ 5 mmol/L) indicators, and number of National Diabetes Audit (NDA) care processes completed (categorised as 0–3, 4–6, or 7–9), in 2010–2011. Outcomes were time to development of DR and sight-threatening diabetic retinopathy (STDR). Nearest neighbour propensity score matching was undertaken and Cox proportional hazards models then fitted using the matched samples. Concordance statistics were calculated for each model.

**Results:**

8263 DR and 832 STDR diagnoses were observed over mean follow-up periods of 3.5 (SD 2.1) and 3.8 (SD 2.0) years, respectively. HbA1c and blood pressure (BP) indicator attainment were associated with lower rates of DR (adjusted hazard ratios (aHRs) 0.94 (95% CI 0.89–0.99) and 0.87 (0.83–0.92), respectively), whereas cholesterol indicator attainment was not (aHR 1.03 (0.97–1.10)). All QOF indicators were associated with lower rates of STDR (aHRs 0.74 (0.62–0.87) for HbA1c, 0.78 (0.67–0.91) for BP, and 0.82 (0.67–0.99) for cholesterol). Completion of 7–9 vs. 0–3 NDA processes was associated with fewer STDR diagnoses (aHR 0.72 (0.55–0.94)).

**Conclusions:**

Attainment of key primary care indicators is associated with lower incidence of DR and STDR among patients with type 2 diabetes in England.

**Supplementary Information:**

The online version contains supplementary material available at 10.1186/s12916-021-01966-x.

## Background

Nearly three million people in England have a type 2 diabetes diagnosis [1–3]. Diabetic retinopathy (DR) is a common complication, affecting nearly a third of patients with type 2 diabetes [4, 5], with considerable impact on quality of life [6]. The severity of DR progresses from mild, moderate, and severe non-proliferative DR (NPDR) to proliferative DR (PDR). Diabetic macular oedema (DME) occurs in about 10% of individuals and can occur in any stage of DR. Sight-threatening DR (STDR) includes severe NPDR, PDR, and DME and is associated with risk of visual loss if not identified early and treated promptly [7, 8]. As DR at all stages is usually asymptomatic prior to visual loss, detection and management of asymptomatic disease is required to halt or slow disease progression and prevent visual loss. Systematic DR screening was introduced in England in 2003 and has been offered to all of the English population since 2008 [9]. The annual uptake was relatively high at 82.8% in 2015–2016 [9].

Further to screening, prevention of DR is in many ways preferable and achievable. Hyperglycaemia and hypertension are understood to be the strongest modifiable risk factors for DR, and controlling these two factors can markedly influence its development and progression. In the UK Prospective Diabetes Study (UKPDS), a 1% lower HbA1c was found to be associated with a 31% lower risk of DR, and a 10 mmHg lower systolic blood pressure (BP) was associated with a 11% lower risk of STDR [10, 11]. Similarly, a recent meta-analysis of four trials showed that a 0.9% lower HbA1c was associated with a 13% lower risk of development and progression of DR [12].

In view of the considerable potential for community-based risk factor control to limit the incidence of diabetes complications—both DR and other complications with important implications for quality of life and life expectancy [13]—England has invested heavily in related quality improvement initiatives over the past two decades. In particular, the Quality and Outcomes Framework (QOF) and National Diabetes Audit (NDA) were introduced in the early 2000s, to incentivise and monitor preventive primary care-based diabetes activities at national level [14, 15]. The vast majority of English general practices participate in QOF, which provides financial awards for achieving specific indicators across a variety of clinical care and public health areas, including diabetes care. NDA uptake was initially limited, but it became a compulsory component of primary care contracts in 2017, and monitors diabetes care provision against the National Institute of Health and Care Excellence (NICE) Clinical Guidelines and associated Quality Standards. The QOF indicators are based on the same guidelines and underlying evidence, and there is therefore some overlap between the two programmes, but it is also the case that the indicators cannot fully reflect the individualised care the guidelines promote [16]. Application of intermediate clinical outcome indicators has required somewhat arbitrary thresholds to be chosen, and despite their prominence in incentivised and mandated national programmes, their association with hard clinical endpoints remain unclear. We therefore aimed here to examine the association between these key clinical outcome indicators and DR, as well as associations between DR and recommended annual diabetes care processes. Specifically, we addressed the questions of whether meeting the QOF HbA1c, BP, and cholesterol thresholds, or completing the NDA care processes, is associated with time to development of DR or STDR, among people with type 2 diabetes.

## Methods

### Study design and data sources

This historical cohort was defined from data extracted from the UK Clinical Practice Research Datalink (CPRD) GOLD database. CPRD GOLD contains longitudinal patient data that have been collected during routine general practice activity since 1987. Presently including more than 18 million patients (3 million of which are currently actively inputting data), the database is representative of the UK primary care registered population. Linked Hospital Episode Statistics (HES) and Office for National Statistics (ONS) mortality data are accessible for CPRD participants in England, and the database has previously been used to assess diabetes care processes and outcomes [17, 18].

Participants entered the cohort on 1 April 2010 so long as they had an existing type 2 diabetes diagnosis, had not opted out of HES/ONS data linkage, were ≥ 18 years old, had been registered with their practice for one or more years, and were not censored prior to 1 April 2011 (for the reasons described below). Those with a DR diagnosis prior to 1 April 2011 were excluded. Those with a type 1 diabetes or other specified non-type 2 diabetes diagnosis at any point were also excluded. Individuals prescribed insulin within three months of a diabetes diagnosis at age ≥ 35 years or within 6 months of a diagnosis made at < 35 years were also excluded. Cohort exit happened at the earliest of the following: death, transfer out of database, last CPRD data upload, or 31 December 2017 (end of study). The code lists used in cohort derivation are provided in Additional File: Table S1.

### Exposures

Attainment of the QOF HbA1c (≤ 7.5% or 59 mmol/mol), BP (≤ 140/80 mmHg), and total cholesterol (≤ 5 mmol/L) indicators within the 2010–2011 financial year was defined according to the QOF Business Rules v38.0 [19]. Thus, the most recent measurements in 2010–2011 were used as the basis for indicator status, and the indicator was considered not to be met where no measurement was available. A further exposure variable describing implementation of NDA annual care processes throughout the 2010–2011 year was created by categorising the number of processes completed as 0–3, 4–6, or 7–9. The NDA care processes include the following: HbA1c, blood pressure, cholesterol, serum creatinine, urine albumin/creatinine ratio, BMI measurements, examination for foot ulcer risk, record of smoking status, and completion of digital retinal screening.

### Outcomes

The primary outcome was time (from 1 April 2011)-to-first CPRD or HES record of incident DR at UK National Screening Committee (NSC) stage R1, R2, R3, M1, or P1 [9]. Time-to-first CPRD or HES record of incident STDR (NSC stage R2, R3, M1, or P1) was considered as a secondary outcome.

### Covariates

Covariates (measured at 1 April 2011) included disease-related variables (time from diagnosis, number of diabetes complications, number of prescribed glucose-lowering therapies (GLTs), and presence/absence of insulin prescription, the latter two measures taken within 6 months prior to baseline), socio-demographic variables (age, sex, ethnicity, 2010 patient-level index of multiple deprivation (IMD)), and the person’s primary care practice geographical region. Comorbidities (number of QOF registers the person was on in 2010–2011, number of hospital admissions in that year, and number of prescriptions during the 6 months prior to cohort entry) and lifestyle variables (body mass index, smoking status, and alcohol use) were also included. A complete list of all covariates can be found in each of Additional File: Tables S2-S17.

### Statistical analysis

Baseline cohort characteristics and missing data were summarised. Practice-level IMD data was used to impute missing patient-level IMD values. Missing lifestyle and ethnicity variables were imputed using the *mice* package in RStudio 3.5.1 from the remaining covariates, with five imputations used [20]. Nearest neighbour propensity score matching was conducted by utilising the *matchit* package with a 0.2 calliper for each exposure [21]. Univariate and multivariate Cox proportional hazards models were fitted with the matched samples for each exposure with the corresponding exposure as another covariate, and concordance statistics were computed for each multivariate model. Sensitivity analyses were conducted for both outcomes to explore the effect of individual QOF indicator attainment among those who had met the other two QOF indicators examined in the study.

## Results

### Cohort characteristics

60,094 adults (44.8% female) registered across 330 practices and diagnosed with type 2 diabetes prior to 1 April 2010 were found as eligible for inclusion. Table 1 presents their baseline characteristics. Mean (standard deviation, SD) age and time since diagnosis was 67.5 (12.7) and 7.1 (5.3) years, respectively. 83.2% were of white ethnic background. Most were current or ex-smokers (52.1%), regularly consumed alcohol at least one unit/week (70.8%), and/or were overweight or obese (83.6%). Individuals had, at baseline, a mean of 2.3 (1.6) comorbidities and 1.4 (1.2) diabetes complications and 7.4 (8.6) different prescriptions including 1.2 (1.0) different GLTs in the 6 months prior to study entry. Insulin was prescribed to 6210 participants (10.3%) throughout that period.

**Table 1:** Baseline characteristics of individuals diagnosed with type 2 diabetes for the population defined within the study period (*N* = 60,094)

Variable	***n*** or mean	% or SD
Age	67.48	12.66
Sex: female	26,893	44.75%
Ethnicity		
Asian	3401	5.66%
Black	1251	2.08%
Mixed	386	0.64%
Other	680	1.13%
White	50,011	83.22%
Missing	4365	7.26%
IMD		
Score	10.25	5.58
Missing	30	0.05%
Region		
North East	1511	2.51%
North West	10,565	17.58%
Yorkshire and The Humber	2310	3.84%
East Midlands	1311	2.18%
West Midlands	7163	11.92%
East of England	6184	10.29%
South West	8112	13.50%
South Central	7352	12.23%
London	7825	13.02%
South East Coast	7761	12.91%
BMI		
Underweight (< 18.5 kg/m^2^)	458	0.76%
Ideal weight (≥ 18.5 to 24.9 kg/m^2^)	8706	14.49%
Overweight (≥ 25.0 to 29.9 kg/m^2^)	19,874	33.07%
Obese (≥ 30.0 kg/m^2^)	30,337	50.48%
Missing	719	1.20%
Never smoker	28,659	47.69%
Ex-smoker	22,540	37.51%
Current smoker	8782	14.61%
Smoking: missing	113	0.19%
Alcohol (units/week)		
0	9714	16.16%
1–14	35,282	58.71%
15–42	5947	9.90%
> 42	1343	2.23%
Missing	7808	12.99%
Number of comorbidities	2.30	1.63
Number of hospitalisations during 2010–2011	0.16	0.52
Duration of diabetes (years)	7.14	5.31
Number of diabetes complications	1.35	1.17
Number of GLT prescriptions within preceding 6 months	1.24	0.98
Insulin prescription within preceding 6 months (Y/N)	6210	10.33%
Number of prescriptions within preceding 6 months	7.43	8.59

*SD*, standard deviation; *IMD*, index of multiple deprivation; *BMI*, body mass index; *GLT*, glucose-lowering therapy

Throughout mean follow-up periods of 3.5 (2.1) and 3.8 (2.0) years, 8263 (13.8%) DR and 832 (1.4%) STDR diagnoses were observed, respectively, corresponding to diagnosis rates of 39.2 and 3.6 per 1000 person-years. The observed distribution of QOF indicator attainment and NDA process completion by the number of indicators/processes met is provided in Table 2. The HbA1c, BP, and cholesterol QOF indicators were met by 40,183 (66.9%), 34,827 (58.0%), and 44,570 (74.2%), respectively. 20,110 (33.5%) participants met all three indicators. NDA process completion ranged between 38,619 (64.3%; retinal screening) and 57,494 (95.7%; BP measurement). The majority (50,284, 83.7%) completed 7–9 NDA processes, though less than half (24,802, 41.3%) completed all nine. Those determined as not attaining the indicator due to lack of available measurement consisted of 3687 (6.1%), 2600 (4.3%), and 5701 (9.5%) individuals for HbA1c, BP, and cholesterol, respectively.

**Table 2:** Number (%) of individuals who met each of the QOF indicators and NDA processes (columns) clustered by the number of QOF indicators or NDA processes met (rows)

Number of indicators/processes met			QOF indicator met			NDA process completed								
		TOTAL	HbA1c	BP	Cholesterol	HbA1c	BP	Cholesterol	Serum creatinine	Urine ACR	Foot exam	BMI	Smoking	Retinal screening
**QOF indicators**	**0**	4293 (7.14%)	0 (0%)	0 (0%)	0 (0%)	2136 (49.76%)	2795 (65.11%)	1826 (42.53%)	2310 (53.81%)	1652 (38.48%)	1968 (45.84%)	2371 (55.23%)	2335 (54.39%)	2103 (48.99%)
	**1**	12,132 (20.19%)	3712 (30.6%)	2980 (24.56%)	5440 (44.84%)	10,917 (89.99%)	11,553 (95.23%)	10,005 (82.47%)	10,859 (89.51%)	8546 (70.44%)	9310 (76.74%)	10,459 (86.21%)	9663 (79.65%)	7459 (61.48%)
	**2**	23,559 (39.2%)	16,361 (69.45%)	11,737 (49.82%)	19,020 (80.73%)	23,244 (98.66%)	23,036 (97.78%)	22,452 (95.3%)	22,973 (97.51%)	18,824 (79.9%)	20,169 (85.61%)	21,722 (92.2%)	20,101 (85.32%)	15,527 (65.91%)
	**3**	20,110 (33.46%)	20,110 (100%)	20,110 (100%)	20,110 (100%)	20,110 (100%)	20,110 (100%)	20,110 (100%)	19,943 (99.17%)	17,111 (85.09%)	18,055 (89.78%)	19,086 (94.91%)	17,668 (87.86%)	13,530 (67.28%)
**NDA processes**	**0–3**	2775 (4.62%)	329 (11.86%)	592 (21.33%)	195 (7.03%)	507 (18.27%)	1202 (43.32%)	286 (10.31%)	598 (21.55%)	120 (4.32%)	201 (7.24%)	353 (12.72%)	507 (18.27%)	853 (30.74%)
	**4–6**	7035 (11.71%)	3957 (56.25%)	3603 (51.22%)	3702 (52.62%)	5851 (83.17%)	6274 (89.18%)	4878 (69.34%)	5634 (80.09%)	2134 (30.33%)	2324 (33.03%)	4014 (57.06%)	3132 (44.52%)	2901 (41.24%)
	**7–9**	50,284 (83.68%)	35,897 (71.39%)	30,632 (60.92%)	40,673 (80.89%)	50,049 (99.53%)	50,018 (99.47%)	49,229 (97.9%)	49,853 (99.14%)	43,879 (87.26%)	46,977 (93.42%)	49,271 (97.99%)	46,128 (91.73%)	34,865 (69.34%)
	**9**	24,802 (41.27%)	18,153 (73.19%)	15,255 (61.51%)	20,961 (84.51%)	24,802 (100%)	24,802 (100%)	24,802 (100%)	24,802 (100%)	24,802 (100%)	24,802 (100%)	24,802 (100%)	24,802 (100%)	24,802 (100%)
**Total**		60,094 (100%)	40,183 (66.87%)	34,827 (57.95%)	44,570 (74.17%)	56,407 (93.86%)	57,494 (95.67%)	54,393 (90.51%)	56,085 (93.33%)	46,133 (76.77%)	49,502 (82.37%)	53,638 (89.26%)	49,767 (82.82%)	38,619 (64.26%)

*QOF*, Quality Outcomes Framework; *NDA*, National Diabetes Audit; *BP*, blood pressure; *ACR*, albumin creatinine ratio

### Associations between QOF indicator exposures and DR and STDR

**Fig. 1:**
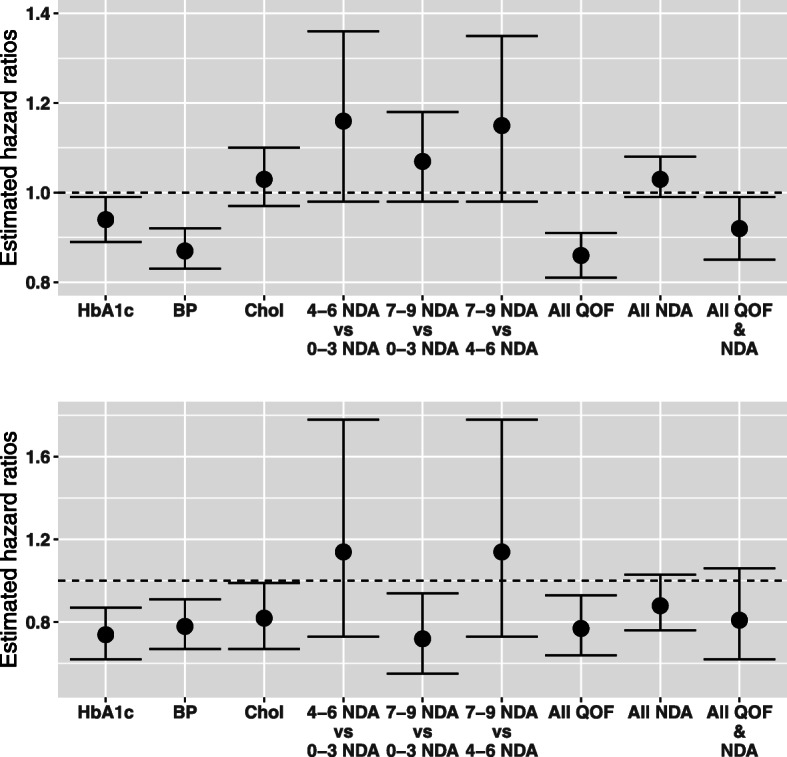
Key adjusted hazard ratio estimates (and corresponding 95% confidence intervals) for risk of DR (top panel) and STDR (bottom panel) across QOF indicator and NDA process exposure definitions

Unadjusted and adjusted associations between exposure to attainment of each of the QOF indicators and incident DR and STDR are shown in Table 3 and adjusted outcomes additionally summarised in Fig. 1. HbA1c and BP indicator attainment were associated with lower rates of DR in both unadjusted and adjusted analyses (adjusted hazard ratios (HRs; 95% CI) 0.94 (0.89–0.99; *P* = 0.030) and 0.87 (0.83–0.92; *P* < 0.001), respectively). No association was observed between cholesterol indicator attainment and DR (adjusted HR 1.03 (0.97–1.10; *P* = 0.292)). HbA1c, BP, and cholesterol QOF indicator attainment were associated with significantly lower rates of STDR in both unadjusted and adjusted analyses (adjusted HR 0.74 (0.62–0.87; *P* < 0.001), 0.78 (0.67–0.91; *P* = 0.002), and 0.82 (0.67–0.99; *P* = 0.043), respectively). Full model results (i.e. including unadjusted and adjusted HR estimates for all covariates) are presented in the Additional File (Tables S2, S4, S5, and S7 for DR and Tables S10, S12, S13, and S15 for STDR outcomes, as well as Figure S1 and S3). C-statistics across multivariate Cox proportional hazards models ranged from 0.75 (95% CI 0.75–0.75) to 0.78 (95% CI 0.78–0.78) across exposures, indicating good fit among all models.

**Table 3:** Unadjusted and adjusted hazard ratios (with corresponding 95% CIs and *p* values) for DR and STDR given QOF exposures after 1:1 propensity score matching, as well as adjusted sample sizes after propensity score matching (*N*) with corresponding outcome events by exposure group

			Outcome events		Unadjusted analyses			Adjusted analyses*		
Outcome	Exposure (indicator met)	***N***	Exposed group	Unexposed group	HR	95% CI	***p***	HR	95% CI	***p***
Retinopathy	HbA1c	37,182	2575	2897	0.87	0.83–0.92	< 0.0001	0.94	0.89–0.99	0.0300
	Blood pressure	50,426	3291	3721	0.88	0.84–0.92	< 0.0001	0.87	0.83–0.92	< 0.0001
	Cholesterol	30,978	2164	2018	1.01	0.95–1.08	0.6988	1.03	0.97–1.10	0.2918
	All QOF indicators	40,220	2402	2773	0.84	0.80–0.89	< 0.0001	0.86	0.81–0.91	< 0.0001
Sight-threatening retinopathy	HbA1c	37,182	235	370	0.66	0.56–0.78	< 0.0001	0.74	0.62–0.87	0.0002
	Blood pressure	50,426	296	400	0.79	0.68–0.92	0.0022	0.78	0.67–0.91	0.0015
	Cholesterol	30,978	200	224	0.80	0.66–0.97	0.0249	0.82	0.67–0.99	0.0428
	All QOF indicators	40,220	191	249	0.75	0.62–0.90	0.0023	0.77	0.64–0.93	0.0065

*HR*, hazard ratio; *CI*, confidence interval; *QOF*, Quality Outcomes Framework. *Adjusted for age, sex, ethnicity, index of multiple deprivation, practice region, body mass index, smoking status, alcohol consumption, number of other co-morbid conditions, hospitalisations, duration of diabetes, diabetes complications, number of glucose-lowering therapies, and insulin prescription status

### Associations between NDA process completion exposures and DR and STDR

Table 4 summarises the associations between the NDA care process categories and incident DR. Point estimates were consistently greater than one (indicating higher rates of DR among those who completed more processes) in both the unadjusted and adjusted analyses. However, evidence for association was low (*p* ≥ 0.079 in all adjusted outcomes). Adjusted HRs are presented graphically in Fig. 1, and full model results are available in the Additional File: Tables S3-S4 and S6-S7 for unadjusted and adjusted results, respectively, as well as Figure S1.

**Table 4:** Unadjusted and adjusted hazard ratios (with corresponding 95% CIs and *p* values) for DR and STDR given NDA exposures after 1:1 propensity score matching, as well as adjusted sample sizes after propensity score matching (*N*) with corresponding outcome events by exposure group

			Outcome events		Unadjusted analyses			Adjusted analyses*		
Outcome	Exposure (processes completed)	***N***	Exposed group	Unexposed group	HR	95% CI	***p***	HR	95% CI	***p***
Retinopathy	4–6 (vs. 0–3)	5438	309	282	1.14	0.97–1.33	0.1165	1.16	0.98–1.36	0.0793
	7–9 (vs. 0–3)	14,052	968	831	1.13	1.03–1.24	0.0097	1.07	0.98–1.18	0.1378
	7–9 (vs. 4–6)	5484	395	289	1.15	0.98–1.34	0.0818	1.15	0.98–1.35	0.0942
	9 (vs. < 9)	49,602	3874	3220	1.02	0.97–1.07	0.4477	1.03	0.99–1.08	0.1703
Sight-threatening retinopathy	4–6 (vs. 0–3)	5438	40	39	1.10	0.71–1.69	0.6834	1.14	0.73–1.78	0.5715
	7–9 (vs. 0–3)	14,052	84	120	0.75	0.57–0.98	0.0325	0.72	0.55–0.94	0.0166
	7–9 (vs. 4–6)	5484	39	39	1.07	0.70–1.63	0.7669	1.14	0.73–1.78	0.5720
	9 (vs. < 9)	49,602	357	351	0.85	0.74–0.99	0.0372	0.88	0.76–1.03	0.1083

*HR*, hazard ratio; *CI*, confidence interval; *adjusted for age, sex, ethnicity, index of multiple deprivation, practice region, body mass index, smoking status, alcohol consumption, number of other co-morbid conditions, hospitalisations, duration of diabetes, diabetes complications, number of glucose-lowering therapies, and insulin prescription status

Unadjusted and adjusted results show evidence of a significant association between completing 7–9 NDA processes (versus 0–3) and development of STDR, with a 28% lower rate of STDR (adjusted HR 0.72 (0.55–0.94; *P* = 0.017)). Additional File (Tables S11-S12 and S14-S15) provides full model results for the secondary outcome, including graphical representations of results (Figure S3).

### Sensitivity analyses

Analyses of the individual QOF indicators among those who had met the other QOF indicators investigated (e.g. HbA1c indicator attainment among those who had met the BP and cholesterol indicators) demonstrated results consistent with our primary analyses for the DR outcome (see Additional File: Tables S8-S9 and Figure S2). For the STDR outcome, evidence of association was observed for only HbA1c indicator attainment (adjusted HR 0.64 (0.49–0.84; *P* = 0.002)) (see Additional File: Tables S16-S17 and Figure S4).

## Discussion

Based on 60,094 people with type 2 diabetes followed up from 2011 to 2017, we observed incidence rates of 39.2 and 3.6 cases per 1000 person-years for DR and STDR, respectively. The corresponding cumulative incidences were 13.8% and 1.4%, respectively. QOF HbA1c and BP indicator attainment was associated with 1–11% and 8–17% lower incidence of DR, respectively, whereas we did not find evidence for association between cholesterol indicator attainment and DR. All three QOF indicators were associated with lower rates of STDR (13–38% lower for HbA1c, 9–33% for BP, and 1–33% for cholesterol). Completing 7–9 NDA processes (versus 0–3) was also associated with a 6–45% lower rate of STDR.

The UKPDS study conducted more than two decades ago on newly diagnosed individuals with type 2 diabetes reported that 22% developed DR and 1.1% required laser photocoagulation (surrogate for STDR) at 6 years [22]. In the Liverpool Diabetes Eye Study (LDES), among 4770 newly diagnosed type 2 diabetes patients between 1991 and 1999, the cumulative incidence of STDR was 3.9% at 5 years [23]. In a cohort study of 16,444 patients with type 2 diabetes from Norwich who were screened from 1990 to 2006, the cumulative incidence of STDR was 0.7% at 5 years and 1.5% at 10 years [24]. The most recent study is another CPRD study that analysed incident DR in type 2 diabetes individuals registered in general practices between 2004 and 2014, in which the age standardised incidence of DR was 40.8 DR/1000 person-years and 9.4 STDR/1000 person-years in 2011 [4]. The differences in these figures with ours could be attributable, at least in part, to the differences in age, study period, follow-up, absolute risk at baseline, and the duration of diabetes between these studies.

In our study, QOF HbA1c indicator attainment was associated with a lower incidence of DR and STDR incidences. The role of HbA1c control in reducing DR progression is unclear. The UKPDS included 3867 newly diagnosed type 2 patients (median age: 54 years), > 95% of whom had no DR or moderate NPDR at baseline [10]. Following intensive glycaemic control therapy with sulphonylureas or insulin, over 10 years of follow-up, the mean HbA1c was 7.0% compared with 7.9% in the conventional treatment group. This was associated with a consistent reduction in DR progression from the sixth year onwards, reaching a relative risk reduction of 21% (0–37%) at about 12 years. In the ACCORD Eye study, which enrolled 2856 participants with type 2 diabetes who were at high risk for cardiovascular disease, the intensive glycaemic control therapy (target HbA1c < 6.0% (42 mmol/mol)) resulted in a significant 23% (13–49%) relative risk reduction in rates of DR progression at 4 years [25]. In contrast, two other RCTs, namely the AdRem [26] and VADT [27] studies, showed no significant effect in reducing DR progression rates with the intensive glycaemic control therapy. In the AdRem study, the difference in median HbA1c between the treatment groups at 4 years was low (< 0.8%), whereas in the VADT study, a large number of individuals had DR with more than moderate severity at baseline. These might likely explain the lack of a significant effect of the intensive glycaemic therapy on DR progression.

With regard to blood pressure, we observed that QOF indicator (≤140/80 mmHg) attainment was associated with  a 13% (3–17%) lower risk of developing DR in people who attained the QOF target of BP ≤ 140/80 mmHg relative to those who did not. Hypertension usually co-exists with diabetes and is another important risk factor for development of DR. The UKPDS study highlighted that intensive control of BP with a target level of 150/85 mmHg or less among hypertensive patients with type 2 diabetes resulted in significant reductions in several aspects of DR after 4.5 years compared to a target of 180/105 mmHg or less [11]. A recent Cochrane review of five RCTs involving 1632 type 2 diabetes patients showed that intense BP control could reduce the incidence of DR by 22% (risk ratio 0.78, 95% CI 0.63; 0.96) compared with less intensive or no intervention over a 4–5-year period [28]. Furthermore, it is important to note that optimising both BP and glycaemic control simultaneously provide an additive effect in renal outcomes and have similar beneficial effects on DR as reported by UKPDS [29].

Our findings related to cholesterol are in line with the FIELD and ACCORD studies. Although these studies showed that either fenofibrate alone or in combination with simvastatin, respectively, reduced the risk of progression to STDR in individuals with type 2 diabetes, but the results did not correlate with the lowering of plasma lipids [25, 30].

A question that may arise is whether the differences in attainment of these key primary care targets are really an accomplishment of the treating physicians or can they be attributed to underlying disease severity or progression. While we may observe differences in number of prescription medications between the exposed and unexposed groups before matching, upon performing propensity score matching, these differences are eliminated (up to the aforementioned calliper) to produce a quasi-randomised equivalent sample (in terms of the covariates of interest). Therefore, we believe the effect seen on STDR is likely due to the attainment of these targets. Similarly, the propensity score matching also helps to alleviate the impact of other potential confounders or mediators such as lifestyle factors. However, causal relationships would be unclear and difficult to demonstrate for mediators, as well as are likely to be heterogeneous both across individuals and covariates.

In relation to the NDA care processes, it is likely that some of these processes are less relevant to DR compared with other diabetes outcomes. However, it is worth noting that, of the nine health checks provided annually to people with type 2 diabetes, the least attained health check was retinal screening at 64.26%, emphasising the need to ensure this is widely offered and accessible.

Our study has several strengths. The sample was large and we were able to account for several important potential confounders in the analyses. The ascertainment of exposures (routine standardised recording) was strong with low levels of missing data [31]. In terms of limitations, incomplete screening and recording will have been associated with under-ascertainment of cases. Some residual confounding is likely, and the study design as related to the care processes limits the interpretation, particularly as some are clearly less directly relevant to DR. There were relatively few STDR cases, which will have limited study power, especially in sensitivity analyses. Finally, we did not account for time-related variation in exposure status, which could have diluted the effect estimates to some extent. Achievement of care processes was measured at baseline (any point during the 1-year baseline period). with only single measurements made, in line with the expectations on primary care providers. We acknowledge that indicator attainment is likely to in part reflect underlying disease severity, as well patient and physician motivations and preferences, as well as the patient-physician relationship. However, we aim to account for the former by making relevant statistical adjustments eg. while we may observe differences in number of prescription medications between the exposed and unexposed groups before matching, upon performing propensity score matching, these differences are eliminated (up to the aforementioned caliper) to produce a quasi-randomized equivalent sample (in terms of the covariates of interest). The latter was also considered in our sensitivity analyses, where the attainment of other QOF indicators was taken into account.

## Conclusions

Overall, our study indicates that attainment of primary care HbA1c and BP indicators is associated with lower incidence of DR and STDR in patients with type 2 diabetes. There is scope to enhance coverage of QOF HbA1c and BP indicator attainment, and thus to potentially limit the incidence of DR and STDR in England, through appropriate community-based measures. Further research is required to examine whether tighter glycaemic and/or BP control could achieve greater reductions in DR incidence without negative impact on macrovascular complications. Moreover, further studies are required to evaluate whether the attainment of these primary care targets will also influence DR progression. 

## Supplementary Information

**Additional file 1: Table S1.** Diabetic retinopathy code list. **Tables S2-S4.** Univariate hazard ratios (with corresponding 95% CIs and *p*-values) for risk of diabetic retinopathy by each covariate across exposure definitions after 1:1 propensity score matching. **Tables S5-S7.** Multivariate hazard ratios (with corresponding 95% CIs and *p*-values) for risk of diabetic retinopathy by each covariate across exposure definitions after 1:1 propensity score matching. **Tables S8-S9.** Univariate and multivariate hazard ratios (with corresponding 95% CIs and *p*-values) for risk of diabetic retinopathy by each covariate across QOF exposure definitions, among those who meet all other QOF targets, after 1:1 propensity score matching. **Tables S10-S12.** Univariate hazard ratios (with corresponding 95% CIs and *p*-values) for risk of sight-threatening diabetic retinopathy by each covariate across exposure definitions after 1:1 propensity score matching. **Tables S13-S15.** Multivariate hazard ratios (with corresponding 95% CIs and p-values) for risk of sight-threatening diabetic retinopathy by each covariate across exposure definitions after 1:1 propensity score matching. **Tables S16-S17.** Univariate and multivariate hazard ratios (with corresponding 95% CIs and p-values) for risk of sight-threatening diabetic retinopathy by each covariate across QOF exposure definitions, among those who meet all other QOF targets, after 1:1 propensity score matching. **Figure S1.** Kaplan-Meier survival curves (and corresponding 95% CIs) for risk of diabetic retinopathy after 1:1 propensity score matching across exposure definitions. **Figure S2.** Kaplan-Meier survival curves (and corresponding 95% CIs) for risk of diabetic retinopathy after 1:1 propensity score matching across QOF exposure definitions, among those who meet all other QOF targets. **Figure S3.** Kaplan-Meier survival curves (and corresponding 95% CIs) for risk of sight-threatening diabetic retinopathy after 1:1 propensity score matching across exposure definitions. **Figure S4.** Kaplan-Meier survival curves (and corresponding 95% CIs) for risk of sight-threatening diabetic retinopathy after 1:1 propensity score matching across QOF exposure definitions, among those who meet all other QOF targets.

## Data Availability

The data that support the findings of this study are available from CPRD but restrictions apply to the availability of these data, which were used under licence for the current study, and so are not publicly available.

## Abbreviations

CPRD: Clinical Practice Research Datalink
QOF: Quality and Outcomes Framework
NDA: National Diabetes Audit
DR: Diabetic retinopathy
STDR: Sight-threatening diabetic retinopathy
aHRs: Adjusted hazard ratios
BP: Blood pressure
DME: Diabetic macular oedema
NPDR: Non-proliferative DR
UKPDS: UK Prospective Diabetes Study
NICE: National Institute of Health and Care Excellence
HES: Hospital Episode Statistics
ONS: Office for National Statistics
LDES: Liverpool Diabetes Eye Study
