# Supplementary material for: Associations between attainment of incentivised primary care indicators and incident diabetic retinopathy in England: a population-based historical cohort study
Source: BMC Med. 2021 Apr 16;19:93. doi: 10.1186/s12916-021-01966-x (PMC8051127; doi:10.1186/s12916-021-01966-x)
Supplement: Supplementary file 1 — Additional file 1: Table S1. Diabetic retinopathy code list. Tables S2-S4. Univariate hazard ratios (with corresponding 95% CIs and p-values) for risk of diabetic retinopathy by each covariate across exposure definitions after 1:1 propensity score matching. Tables S5-S7. Multivariate hazard ratios (with corresponding 95% CIs and p-values) for risk of diabetic retinopathy by each covariate across exposure definitions after 1:1 propensity score matching. Tables S8-S9. Univariate and multivariate hazard ratios (with corresponding 95% CIs and p-values) for risk of diabetic retinopathy by each covariate across QOF exposure definitions, among those who meet all other QOF targets, after 1:1 propensity score matching. Tables S10-S12. Univariate hazard ratios (with corresponding 95% CIs and p-values) for risk of sight-threatening diabetic retinopathy by each covariate across exposure definitions after 1:1 propensity score matching. Tables S13-S15. Multivariate hazard ratios (with corresponding 95% CIs and p-values) for risk of sight-threatening diabetic retinopathy by each covariate across exposure definitions after 1:1 propensity score matching. Tables S16-S17. Univariate and multivariate hazard ratios (with corresponding 95% CIs and p-values) for risk of sight-threatening diabetic retinopathy by each covariate across QOF exposure definitions, among those who meet all other QOF targets, after 1:1 propensity score matching. Figure S1. Kaplan-Meier survival curves (and corresponding 95% CIs) for risk of diabetic retinopathy after 1:1 propensity score matching across exposure definitions. Figure S2. Kaplan-Meier survival curves (and corresponding 95% CIs) for risk of diabetic retinopathy after 1:1 propensity score matching across QOF exposure definitions, among those who meet all other QOF targets. Figure S3. Kaplan-Meier survival curves (and corresponding 95% CIs) for risk of sight-threatening diabetic retinopathy after 1:1 propensity score matching across exposure definition [file 12916_2021_1966_MOESM1_ESM.docx]

Additional File 1: Table S1: Diabetic retinopathy code list.

| **medcode** | **Read code** | **Read term** |
| --- | --- | --- |
| 22967 | 2BBf.00 | Retinal abnormality - diabetes related |
| 11433 | 2BBP.00 | O/E - right eye background diabetic retinopathy |
| 11129 | 2BBQ.00 | O/E - left eye background diabetic retinopathy |
| 11018 | 8HBG.00 | Diabetic retinopathy 12 month review |
| 17262 | C109600 | Non-insulin-dependent diabetes mellitus with retinopathy |
| 58604 | C109611 | Type II diabetes mellitus with retinopathy |
| 42762 | C109612 | Type 2 diabetes mellitus with retinopathy |
| 18496 | C10F600 | Type 2 diabetes mellitus with retinopathy |
| 49655 | C10F611 | Type II diabetes mellitus with retinopathy |
| 1323 | F420.00 | Diabetic retinopathy |
| 7069 | F420000 | Background diabetic retinopathy |
| 10755 | F420600 | Non proliferative diabetic retinopathy |
| 11626 | F420z00 | Diabetic retinopathy NOS |
| 101881 | 2BBr.00 | Impaired vision due to diabetic retinopathy |
| 13099 | 2BBR.00 | O/E - right eye preproliferative diabetic retinopathy |
| 13103 | 2BBS.00 | O/E - left eye preproliferative diabetic retinopathy |
| 18662 | 8HBH.00 | Diabetic retinopathy 6 month review |
| 2986 | F420200 | Preproliferative diabetic retinopathy |
| 47584 | F420500 | Advanced diabetic retinal disease |
| 47328 | 2BBk.00 | O/E - right eye stable treated prolif diabetic retinopathy |
| 52041 | 2BBl.00 | O/E - left eye stable treated prolif diabetic retinopathy |
| 11599 | 7276 | Pan retinal photocoagulation for diabetes |
| 13097 | 2BBT.00 | O/E - right eye proliferative diabetic retinopathy |
| 13101 | 2BBV.00 | O/E - left eye proliferative diabetic retinopathy |
| 3286 | F420100 | Proliferative diabetic retinopathy |
| 30477 | F420700 | High risk proliferative diabetic retinopathy |
| 65463 | F420800 | High risk non proliferative diabetic retinopathy |
| 9835 | 2BBL.00 | O/E - diabetic maculopathy present both eyes |
| 52630 | 2BBo.00 | O/E - sight threatening diabetic retinopathy |
| 13102 | 2BBW.00 | O/E - right eye diabetic maculopathy |
| 13108 | 2BBX.00 | O/E - left eye diabetic maculopathy |
| 25591 | C10FQ00 | Type 2 diabetes mellitus with exudative maculopathy |
| 111798 | C10FQ11 | Type II diabetes mellitus with exudative maculopathy |
| 10099 | F420300 | Advanced diabetic maculopathy |
| 3837 | F420400 | Diabetic maculopathy |
| 19533 | 2BBY.00 | O/E - referable retinopathy |
| 39457 | F421C00 | Other intraretinal microvascular abnormality |
| 3822 | 2BB8.00 | O/E - vitreous haemorrhages |
| 25888 | 2BBm.00 | O/E - right eye clinically significant macular oedema |
| 13107 | 2BBn.00 | O/E - left eye clinically significant macular oedema |
| 100979 | 7272900 | Focal laser photocoagulation of retina |
| 33681 | 2BB..00 | O/E - retinal inspection |
| 24080 | 2BB1.00 | O/E - retina normal |
| 17198 | 2BB..11 | O/E - retina |
| 45145 | 2BB2.00 | O/E - retinal vessel narrowing |
| 48751 | 2BB3.00 | O/E - retinal A-V nipping |
| 19532 | 2BB4.00 | O/E - retinal microaneurysms |
| 8742 | 2BB5.00 | O/E - retinal haemorrhages |
| 13106 | 2BB6.00 | O/E - retinal exudates |
| 35659 | 2BB7.00 | O/E - retinal vascular prolif. |
| 3822 | 2BB8.00 | O/E - vitreous haemorrhages |
| 3914 | 2BB9.00 | O/E - retinal pigmentation |
| 36867 | 2BBa.00 | O/E- non-referable retinopathy |
| 19535 | 2BBA.00 | Examination of retina |
| 50656 | 2BBc.00 | O/E - No retinal laser photocoagulation scars |
| 31088 | 2BBD.00 | O/E - Right retina normal |
| 70163 | 2BBe.00 | O/E - right retina partially assessable |
| 31089 | 2BBE.00 | O/E - Left retina normal |
| 66273 | 2BBg.00 | O/E - right retina fully assessable |
| 26538 | 2BBi.00 | O/E - right eye no maculopathy |
| 19149 | 2BBI.00 | O/E - no retinopathy |
| 43501 | 2BBj.00 | O/E - left eye no maculopathy |
| 13100 | 2BBJ.00 | O/E - no right diabetic retinopathy |
| 47328 | 2BBk.00 | O/E - right eye stable treated prolif diabetic retinopathy |
| 13104 | 2BBK.00 | O/E - no left diabetic retinopathy |
| 52041 | 2BBl.00 | O/E - left eye stable treated prolif diabetic retinopathy |
| 9835 | 2BBL.00 | O/E - diabetic maculopathy present both eyes |
| 25888 | 2BBm.00 | O/E - right eye clinically significant macular oedema |
| 47144 | 2BBM.00 | O/E - diabetic maculopathy absent both eyes |
| 13107 | 2BBn.00 | O/E - left eye clinically significant macular oedema |
| 52630 | 2BBo.00 | O/E - sight threatening diabetic retinopathy |
| 18775 | 2BBO.00 | O/E - Laser photocoagulation scars |
| 11433 | 2BBP.00 | O/E - right eye background diabetic retinopathy |
| 11129 | 2BBQ.00 | O/E - left eye background diabetic retinopathy |
| 13099 | 2BBR.00 | O/E - right eye preproliferative diabetic retinopathy |
| 13103 | 2BBS.00 | O/E - left eye preproliferative diabetic retinopathy |
| 13097 | 2BBT.00 | O/E - right eye proliferative diabetic retinopathy |
| 13101 | 2BBV.00 | O/E - left eye proliferative diabetic retinopathy |
| 13102 | 2BBW.00 | O/E - right eye diabetic maculopathy |
| 13108 | 2BBX.00 | O/E - left eye diabetic maculopathy |
| 19533 | 2BBY.00 | O/E - referable retinopathy |
| 25116 | 2BBZ.00 | O/E - retinal inspection NOS |
| 878 | 3128000 | Fundoscopy normal |
| 1411 | 3128100 | Fundoscopy abnormal |
| 19531 | 3128.11 | Retinoscopy |
| 21754 | 3128200 | Dilated fundoscopy normal |
| 19534 | 3128300 | Camera fundoscopy |
| 22966 | 3128400 | Indirect fundoscopy following mydriatic |
| 13098 | 3128Z00 | Fundoscopy NOS |
| 17871 | 312E.00 | Direct fundoscopy following mydriatic |
| 64070 | 312G.00 | Indirect fundoscopy following mydriatic |
| 13105 | 58C1.00 | Retinal photography |
| 13196 | 66AD.00 | Fundoscopy - diabetic check |
| 18311 | 68A7.00 | Diabetic retinopathy screening |
| 11891 | 68A8.00 | Digital retinal screening |
| 881 | 3128 | Fundoscopy |
| 92317 | 2BBf.00 | O/E - left retina partially assessable |
| 36619 | 312F.00 | Camera fundoscopy |
| 30111 | 3129 | Eye fundus photography |
| 20991 | 312A.00 | Slit lamp examination |
| 95916 | 2BBH.00 | O/E - left retina fully assessable |

Additional File 1: Table S2: Univariate hazard ratios (with corresponding 95% CIs and p-values) for risk of diabetic retinopathy by each covariate across QOF exposure definitions after 1:1 propensity score matching.

|  | **Exposure Definition** | | | | | | | | | | |
| --- | --- | --- | --- | --- | --- | --- | --- | --- | --- | --- | --- |
|  | **Achieve HbA1c QOF**  **Target** | | |  | **Achieve Blood Pressure QOF Target** | | |  | **Achieve Cholesterol QOF**  **Target** | | |
|  | **Hazard Ratio** | **95% CI** | **p** |  | **Hazard Ratio** | **95% CI** | **p** |  | **Hazard Ratio** | **95% CI** | **p** |
| ***Exposure*** | 0.87 | 0.83-0.92 | <0.0001 |  | 0.88 | 0.84-0.92 | <0.0001 |  | 1.01 | 0.95-1.08 | 0.6988 |
| ***Age*** | 1.00 | 1.00-1.00 | 0.6553 |  | 1.00 | 1.00-1.00 | 0.4843 |  | 1.00 | 0.99-1.00 | 0.0075 |
| ***Sex: Female*** | 0.94 | 0.89-0.99 | 0.0157 |  | 0.93 | 0.89-0.98 | 0.0034 |  | 0.91 | 0.86-0.97 | 0.0025 |
| ***Ethnicity: Asian*** | 0.96 | 0.87-1.06 | 0.4278 |  | 1.00 | 0.91-1.10 | 0.9304 |  | 1.00 | 0.88-1.13 | 0.9927 |
| ***Ethnicity: Black*** | 0.99 | 0.85-1.17 | 0.9484 |  | 1.01 | 0.88-1.17 | 0.8455 |  | 1.12 | 0.95-1.34 | 0.1844 |
| ***Ethnicity: Mixed*** | 1.69 | 1.32-2.16 | <0.0001 |  | 1.63 | 1.32-2.02 | <0.0001 |  | 1.71 | 1.28-2.28 | 0.0003 |
| ***Ethnicity: Other*** | 0.96 | 0.75-1.22 | 0.7162 |  | 0.93 | 0.75-1.16 | 0.5192 |  | 0.79 | 0.59-1.05 | 0.1008 |
| ***IMD*** | 1.00 | 0.99-1.00 | 0.5283 |  | 1.00 | 0.99-1.00 | 0.4403 |  | 1.00 | 0.99-1.00 | 0.3442 |
| ***North West*** | 0.95 | 0.89-1.02 | 0.1890 |  | 0.97 | 0.91-1.03 | 0.3675 |  | 1.04 | 0.96-1.12 | 0.3410 |
| ***Yorkshire & Humber*** | 1.14 | 1.00-1.29 | 0.0549 |  | 1.09 | 0.97-1.22 | 0.1552 |  | 0.98 | 0.83-1.15 | 0.7985 |
| ***East Midlands*** | 0.98 | 0.82-1.18 | 0.8473 |  | 1.01 | 0.86-1.19 | 0.9038 |  | 1.02 | 0.83-1.25 | 0.8659 |
| ***West Midlands*** | 1.02 | 0.94-1.11 | 0.5602 |  | 1.02 | 0.95-1.10 | 0.5457 |  | 1.05 | 0.95-1.15 | 0.3512 |
| ***East of England*** | 0.93 | 0.85-1.02 | 0.1250 |  | 0.97 | 0.90-1.05 | 0.4685 |  | 0.96 | 0.87-1.07 | 0.4774 |
| ***South West*** | 0.99 | 0.92-1.07 | 0.8709 |  | 1.05 | 0.98-1.12 | 0.1780 |  | 0.99 | 0.91-1.09 | 0.8882 |
| ***South Central*** | 1.06 | 0.98-1.14 | 0.1793 |  | 1.03 | 0.95-1.10 | 0.4940 |  | 1.04 | 0.94-1.14 | 0.4596 |
| ***London*** | 1.05 | 0.97-1.13 | 0.2447 |  | 1.02 | 0.95-1.09 | 0.6343 |  | 0.98 | 0.90-1.08 | 0.7382 |
| ***South East Coast*** | 0.97 | 0.90-1.05 | 0.4476 |  | 0.93 | 0.86-1.00 | 0.0406 |  | 0.92 | 0.84-1.01 | 0.0783 |
| ***BMI: Underweight*** | 1.01 | 0.93-1.09 | 0.8445 |  | 1.01 | 0.94-1.09 | 0.7297 |  | 0.96 | 0.88-1.05 | 0.3482 |
| ***BMI: Overweight*** | 1.07 | 1.01-1.13 | 0.0164 |  | 1.05 | 1.00-1.11 | 0.0463 |  | 1.07 | 1.00-1.14 | 0.0356 |
| ***BMI: Obese*** | 0.95 | 0.90-1.00 | 0.0717 |  | 0.97 | 0.92-1.02 | 0.1886 |  | 0.98 | 0.92-1.04 | 0.5492 |
| ***BMI: Missing*** | 0.72 | 0.55-0.94 | 0.0165 |  | 0.70 | 0.54-0.90 | 0.0048 |  | 0.65 | 0.48-0.89 | 0.0077 |
| ***Ex-Smoker*** | 1.03 | 0.97-1.09 | 0.3194 |  | 1.00 | 0.96-1.05 | 0.8576 |  | 1.05 | 0.98-1.12 | 0.1599 |
| ***Current Smoker*** | 0.95 | 0.88-1.02 | 0.1393 |  | 0.98 | 0.91-1.05 | 0.5083 |  | 0.97 | 0.89-1.05 | 0.4036 |
| ***Smoking: Missing*** | 2.32 | 1.60-3.36 | <0.0001 |  | 3.03 | 2.17-4.22 | <0.0001 |  | 2.34 | 1.52-3.59 | 0.0001 |
| ***Alcohol: 1-14*** | 0.94 | 0.89-0.99 | 0.0235 |  | 0.94 | 0.90-0.99 | 0.0101 |  | 0.93 | 0.87-0.98 | 0.0140 |
| ***Alcohol: 15-42*** | 1.04 | 0.96-1.14 | 0.3410 |  | 1.04 | 0.97-1.13 | 0.2765 |  | 1.13 | 1.02-1.24 | 0.0182 |
| ***Alcohol: >42*** | 1.01 | 0.84-1.21 | 0.8955 |  | 0.93 | 0.80-1.10 | 0.4060 |  | 0.86 | 0.71-1.06 | 0.1611 |
| ***Alcohol: Missing*** | 0.89 | 0.82-0.96 | 0.0027 |  | 0.89 | 0.83-0.96 | 0.0021 |  | 0.86 | 0.79-0.95 | 0.0018 |
| ***Morbidities*** | 0.98 | 0.97-1.00 | 0.0502 |  | 1.00 | 0.98-1.01 | 0.5727 |  | 0.99 | 0.97-1.01 | 0.2850 |
| ***Prescriptions*** | 1.01 | 1.00-1.01 | <0.0001 |  | 1.01 | 1.01-1.01 | <0.0001 |  | 1.01 | 1.01-1.01 | <0.0001 |
| ***Hospitalisations*** | 1.01 | 0.96-1.07 | 0.6099 |  | 1.03 | 0.98-1.08 | 0.2988 |  | 1.02 | 0.96-1.08 | 0.5532 |
| ***Duration of diabetes (years)*** | 1.00 | 1.00-1.01 | 0.4999 |  | 1.00 | 1.00-1.01 | 0.1127 |  | 1.01 | 1.00-1.01 | 0.0243 |
| ***Complications*** | 1.70 | 1.67-1.73 | <0.0001 |  | 1.72 | 1.69-1.75 | <0.0001 |  | 1.75 | 1.71-1.79 | <0.0001 |
| ***Glucose lowering therapies*** | 1.18 | 1.15-1.21 | <0.0001 |  | 1.22 | 1.19-1.24 | <0.0001 |  | 1.25 | 1.21-1.28 | <0.0001 |
| ***Insulin prescription*** | 1.40 | 1.30-1.50 | <0.0001 |  | 1.58 | 1.47-1.69 | <0.0001 |  | 1.68 | 1.54-1.83 | <0.0001 |

Study sizes across exposures after 1:1 propensity score matching are found in Supplementary Table 4, as they are the same between univariate and multivariate analyses.

Additional File 1: Table S3: Univariate hazard ratios (with corresponding 95% CIs and p-values) for risk of diabetic retinopathy by each covariate across NDA exposure definitions after 1:1 propensity score matching.

|  | **Exposure Definition** | | | | | | | | | | |
| --- | --- | --- | --- | --- | --- | --- | --- | --- | --- | --- | --- |
|  | **Meet 4-6 NDA Processes (vs. Meet 0-3 NDA Processes)** | | |  | **Meet 7-9 NDA Processes (vs. Meet 0-3 NDA Processes)** | | |  | **Meet 7-9 NDA Processes (vs. Meet 4-6 NDA Processes)** | | |
|  | **Hazard Ratio** | **95% CI** | **p** |  | **Hazard Ratio** | **95% CI** | **p** |  | **Hazard Ratio** | **95% CI** | **p** |
| ***Exposure*** | 1.14 | 0.97-1.33 | 0.1165 |  | 1.13 | 1.03-1.24 | 0.0097 |  | 1.15 | 0.98-1.34 | 0.0818 |
| ***Age*** | 0.99 | 0.99-1.00 | 0.0271 |  | 1.00 | 0.99-1.00 | 0.1430 |  | 1.00 | 0.99-1.00 | 0.1456 |
| ***Sex: Female*** | 0.85 | 0.73-1.00 | 0.0552 |  | 0.91 | 0.83-1.00 | 0.0418 |  | 0.93 | 0.79-1.09 | 0.3562 |
| ***Ethnicity: Asian*** | 1.11 | 0.83-1.47 | 0.4927 |  | 0.95 | 0.80-1.13 | 0.5694 |  | 0.89 | 0.64-1.23 | 0.4732 |
| ***Ethnicity: Black*** | 0.89 | 0.59-1.34 | 0.5908 |  | 1.06 | 0.83-1.35 | 0.6451 |  | 1.18 | 0.82-1.72 | 0.3756 |
| ***Ethnicity: Mixed*** | 2.98 | 1.72-5.16 | 0.0001 |  | 2.05 | 1.34-3.11 | 0.0009 |  | 1.78 | 0.95-3.32 | 0.0704 |
| ***Ethnicity: Other*** | 0.82 | 0.42-1.58 | 0.5545 |  | 0.80 | 0.52-1.24 | 0.3218 |  | 1.33 | 0.80-2.22 | 0.2782 |
| ***IMD*** | 0.99 | 0.98-1.01 | 0.2256 |  | 1.00 | 0.99-1.01 | 0.8304 |  | 0.99 | 0.97-1.00 | 0.0472 |
| ***North West*** | 0.86 | 0.69-1.07 | 0.1794 |  | 1.02 | 0.90-1.15 | 0.7497 |  | 0.81 | 0.65-1.01 | 0.0593 |
| ***Yorkshire & Humber*** | 1.56 | 1.10-2.22 | 0.0130 |  | 1.15 | 0.91-1.44 | 0.2438 |  | 1.38 | 0.94-2.01 | 0.0982 |
| ***East Midlands*** | 0.97 | 0.58-1.62 | 0.9098 |  | 1.08 | 0.79-1.47 | 0.6434 |  | 0.89 | 0.54-1.47 | 0.6542 |
| ***West Midlands*** | 1.20 | 0.95-1.50 | 0.1231 |  | 1.05 | 0.92-1.21 | 0.4629 |  | 1.12 | 0.89-1.40 | 0.3350 |
| ***East of England*** | 0.98 | 0.75-1.29 | 0.9074 |  | 0.90 | 0.77-1.05 | 0.1655 |  | 1.21 | 0.96-1.52 | 0.1123 |
| ***South West*** | 0.83 | 0.64-1.06 | 0.1335 |  | 0.90 | 0.78-1.03 | 0.1296 |  | 0.97 | 0.77-1.21 | 0.7789 |
| ***South Central*** | 1.23 | 0.99-1.54 | 0.0667 |  | 1.08 | 0.94-1.24 | 0.2537 |  | 0.94 | 0.73-1.20 | 0.5943 |
| ***London*** | 0.90 | 0.71-1.15 | 0.3979 |  | 1.15 | 1.01-1.32 | 0.0299 |  | 0.98 | 0.78-1.24 | 0.8646 |
| ***South East Coast*** | 0.98 | 0.77-1.25 | 0.8670 |  | 0.85 | 0.74-0.98 | 0.0293 |  | 1.03 | 0.82-1.29 | 0.8070 |
| ***BMI: Underweight*** | 0.86 | 0.67-1.09 | 0.2097 |  | 1.03 | 0.91-1.18 | 0.6089 |  | 1.09 | 0.88-1.36 | 0.4345 |
| ***BMI: Overweight*** | 1.13 | 0.96-1.34 | 0.1503 |  | 1.03 | 0.94-1.14 | 0.5180 |  | 1.14 | 0.97-1.35 | 0.1140 |
| ***BMI: Obese*** | 0.98 | 0.83-1.15 | 0.7803 |  | 1.01 | 0.92-1.10 | 0.9086 |  | 0.86 | 0.74-1.01 | 0.0621 |
| ***BMI: Missing*** | 0.54 | 0.29-1.00 | 0.0511 |  | 0.44 | 0.27-0.71 | 0.0007 |  | 0.75 | 0.46-1.21 | 0.2358 |
| ***Ex-Smoker*** | 1.08 | 0.91-1.28 | 0.4049 |  | 1.06 | 0.97-1.17 | 0.2080 |  | 1.13 | 0.96-1.33 | 0.1563 |
| ***Current Smoker*** | 0.82 | 0.66-1.01 | 0.0654 |  | 0.96 | 0.84-1.09 | 0.5258 |  | 0.78 | 0.63-0.95 | 0.0159 |
| ***Smoking: Missing*** | 2.49 | 1.18-5.25 | 0.0164 |  | 2.29 | 1.30-4.04 | 0.0042 |  | 4.60 | 2.54-8.35 | <0.0001 |
| ***Alcohol: 1-14*** | 0.94 | 0.80-1.11 | 0.4689 |  | 0.95 | 0.87-1.05 | 0.3260 |  | 1.03 | 0.89-1.21 | 0.6641 |
| ***Alcohol: 15-42*** | 1.18 | 0.93-1.50 | 0.1800 |  | 1.11 | 0.95-1.29 | 0.1910 |  | 0.94 | 0.73-1.20 | 0.6044 |
| ***Alcohol: >42*** | 1.04 | 0.67-1.61 | 0.8529 |  | 1.11 | 0.85-1.46 | 0.4326 |  | 0.64 | 0.39-1.06 | 0.0825 |
| ***Alcohol: Missing*** | 0.83 | 0.67-1.03 | 0.0857 |  | 0.85 | 0.75-0.97 | 0.0193 |  | 0.98 | 0.80-1.20 | 0.8247 |
| ***Morbidities*** | 0.96 | 0.91-1.02 | 0.1639 |  | 0.97 | 0.95-1.00 | 0.0863 |  | 0.95 | 0.90-1.00 | 0.0367 |
| ***Prescriptions*** | 1.00 | 1.00-1.01 | 0.1969 |  | 1.01 | 1.00-1.01 | 0.0052 |  | 1.01 | 1.00-1.01 | 0.0326 |
| ***Hospitalisations*** | 0.89 | 0.75-1.06 | 0.1890 |  | 0.99 | 0.91-1.07 | 0.7415 |  | 1.02 | 0.89-1.18 | 0.7418 |
| ***Duration of diabetes (years)*** | 1.02 | 1.00-1.03 | 0.0070 |  | 1.01 | 1.00-1.01 | 0.1959 |  | 1.01 | 1.00-1.03 | 0.0799 |
| ***Complications*** | 1.76 | 1.66-1.87 | <0.0001 |  | 1.71 | 1.65-1.77 | <0.0001 |  | 1.75 | 1.65-1.84 | <0.0001 |
| ***Glucose lowering therapies*** | 1.35 | 1.26-1.45 | <0.0001 |  | 1.23 | 1.18-1.29 | <0.0001 |  | 1.27 | 1.19-1.35 | <0.0001 |
| ***Insulin prescription*** | 1.81 | 1.43-2.29 | <0.0001 |  | 1.58 | 1.39-1.78 | <0.0001 |  | 1.75 | 1.40-2.18 | <0.0001 |

Study sizes across exposures after 1:1 propensity score matching are found in Supplementary Table 5, as they are the same between univariate and multivariate analyses.

Additional File 1: Table S4: Univariate hazard ratios (with corresponding 95% CIs and p-values) for risk of diabetic retinopathy by each covariate across NDA and QOF exposure definitions after 1:1 propensity score matching.

|  | **Exposure Definition** | | | | | | | | | | |
| --- | --- | --- | --- | --- | --- | --- | --- | --- | --- | --- | --- |
|  | **Achieve All QOF Targets** | | |  | **Meet All NDA Processes** | | |  | **Achieve All QOF & NDA Targets** | | |
|  | **Hazard Ratio** | **95% CI** | **p** |  | **Hazard Ratio** | **95% CI** | **p** |  | **Hazard Ratio** | **95% CI** | **p** |
| ***Exposure*** | 0.84 | 0.80-0.89 | <0.0001 |  | 1.02 | 0.97-1.07 | 0.4477 |  | 0.88 | 0.82-0.95 | 0.0014 |
| ***Age*** | 1.00 | 1.00-1.00 | 0.0339 |  | 1.00 | 1.00-1.00 | 0.1016 |  | 1.00 | 1.00-1.01 | 0.0046 |
| ***Sex: Female*** | 0.95 | 0.90-1.01 | 0.0996 |  | 0.96 | 0.92-1.01 | 0.1174 |  | 0.93 | 0.86-1.01 | 0.0732 |
| ***Ethnicity: Asian*** | 0.93 | 0.83-1.05 | 0.2380 |  | 0.98 | 0.89-1.08 | 0.7008 |  | 0.85 | 0.71-1.02 | 0.0847 |
| ***Ethnicity: Black*** | 1.03 | 0.83-1.26 | 0.8143 |  | 0.99 | 0.83-1.18 | 0.9192 |  | 0.88 | 0.62-1.25 | 0.4809 |
| ***Ethnicity: Mixed*** | 1.47 | 1.09-1.98 | 0.0123 |  | 1.49 | 1.19-1.87 | 0.0005 |  | 1.47 | 0.99-2.20 | 0.0587 |
| ***Ethnicity: Other*** | 0.90 | 0.68-1.18 | 0.4385 |  | 0.82 | 0.64-1.05 | 0.1228 |  | 1.02 | 0.71-1.48 | 0.9016 |
| ***IMD*** | 1.00 | 0.99-1.00 | 0.5701 |  | 1.00 | 0.99-1.00 | 0.5358 |  | 1.00 | 0.99-1.00 | 0.3421 |
| ***North West*** | 0.99 | 0.92-1.06 | 0.8235 |  | 0.99 | 0.93-1.05 | 0.6733 |  | 1.01 | 0.91-1.11 | 0.8965 |
| ***Yorkshire & Humber*** | 1.01 | 0.88-1.16 | 0.8778 |  | 1.06 | 0.94-1.20 | 0.3229 |  | 0.94 | 0.77-1.15 | 0.5616 |
| ***East Midlands*** | 0.97 | 0.81-1.17 | 0.7813 |  | 1.01 | 0.86-1.18 | 0.9278 |  | 0.78 | 0.58-1.04 | 0.0904 |
| ***West Midlands*** | 1.04 | 0.96-1.13 | 0.3021 |  | 1.01 | 0.94-1.08 | 0.8153 |  | 1.06 | 0.94-1.18 | 0.3385 |
| ***East of England*** | 0.97 | 0.88-1.06 | 0.4707 |  | 0.98 | 0.91-1.06 | 0.6853 |  | 0.94 | 0.83-1.07 | 0.3405 |
| ***South West*** | 1.02 | 0.94-1.10 | 0.6646 |  | 1.00 | 0.93-1.07 | 0.9474 |  | 1.04 | 0.93-1.16 | 0.5047 |
| ***South Central*** | 1.10 | 1.01-1.19 | 0.0233 |  | 1.04 | 0.97-1.12 | 0.2463 |  | 1.08 | 0.96-1.21 | 0.1919 |
| ***London*** | 1.00 | 0.92-1.08 | 0.9122 |  | 1.03 | 0.96-1.10 | 0.3816 |  | 1.03 | 0.92-1.15 | 0.5763 |
| ***South East Coast*** | 0.89 | 0.82-0.97 | 0.0091 |  | 0.94 | 0.88-1.01 | 0.0852 |  | 0.89 | 0.79-1.01 | 0.0662 |
| ***BMI: Underweight*** | 1.05 | 0.98-1.13 | 0.1727 |  | 1.01 | 0.94-1.08 | 0.8577 |  | 1.05 | 0.95-1.16 | 0.3642 |
| ***BMI: Overweight*** | 1.07 | 1.01-1.13 | 0.0231 |  | 1.04 | 0.99-1.10 | 0.0915 |  | 1.06 | 0.98-1.14 | 0.1474 |
| ***BMI: Obese*** | 0.93 | 0.88-0.98 | 0.0106 |  | 0.97 | 0.93-1.02 | 0.2588 |  | 0.94 | 0.87-1.01 | 0.1128 |
| ***BMI: Missing*** | 0.68 | 0.47-0.98 | 0.0405 |  | 0.72 | 0.53-0.96 | 0.0250 |  | 0.48 | 0.25-0.93 | 0.0298 |
| ***Ex-Smoker*** | 1.03 | 0.97-1.08 | 0.3717 |  | 0.98 | 0.93-1.03 | 0.4080 |  | 1.03 | 0.95-1.11 | 0.4450 |
| ***Current Smoker*** | 0.99 | 0.91-1.07 | 0.7468 |  | 1.02 | 0.95-1.08 | 0.6593 |  | 1.02 | 0.91-1.14 | 0.7494 |
| ***Smoking: Missing*** | 2.64 | 1.50-4.66 | 0.0008 |  | N/A | N/A | N/A |  | N/A | N/A | N/A |
| ***Alcohol: 1-14*** | 0.96 | 0.91-1.02 | 0.1534 |  | 0.92 | 0.88-0.97 | 0.0011 |  | 1.02 | 0.94-1.10 | 0.7036 |
| ***Alcohol: 15-42*** | 1.03 | 0.95-1.13 | 0.4640 |  | 1.03 | 0.96-1.12 | 0.3813 |  | 0.84 | 0.73-0.96 | 0.0089 |
| ***Alcohol: >42*** | 0.97 | 0.80-1.18 | 0.7728 |  | 0.94 | 0.80-1.12 | 0.4884 |  | 0.88 | 0.66-1.16 | 0.3664 |
| ***Alcohol: Missing*** | 0.82 | 0.75-0.90 | <0.0001 |  | 0.88 | 0.82-0.95 | 0.0019 |  | 0.86 | 0.76-0.99 | 0.0317 |
| ***Morbidities*** | 1.00 | 0.99-1.02 | 0.6612 |  | 0.98 | 0.97-1.00 | 0.0465 |  | 0.99 | 0.96-1.01 | 0.2893 |
| ***Prescriptions*** | 1.01 | 1.01-1.01 | <0.0001 |  | 1.01 | 1.01-1.01 | <0.0001 |  | 1.01 | 1.01-1.02 | <0.0001 |
| ***Hospitalisations*** | 1.00 | 0.94-1.06 | 0.9530 |  | 1.03 | 0.97-1.09 | 0.2896 |  | 1.02 | 0.93-1.13 | 0.6266 |
| ***Duration of diabetes (years)*** | 1.00 | 1.00-1.01 | 0.6434 |  | 1.00 | 1.00-1.01 | 0.5280 |  | 1.00 | 0.99-1.01 | 0.9395 |
| ***Complications*** | 1.73 | 1.70-1.77 | <0.0001 |  | 1.69 | 1.67-1.72 | <0.0001 |  | 1.73 | 1.68-1.78 | <0.0001 |
| ***Glucose lowering therapies*** | 1.21 | 1.18-1.25 | <0.0001 |  | 1.20 | 1.18-1.23 | <0.0001 |  | 1.22 | 1.18-1.27 | <0.0001 |
| ***Insulin prescription*** | 1.65 | 1.50-1.81 | <0.0001 |  | 1.53 | 1.43-1.63 | <0.0001 |  | 1.65 | 1.44-1.90 | <0.0001 |

Study sizes across exposures after 1:1 propensity score matching are found in Supplementary Table 6, as they are the same between univariate and multivariate analyses.

N/A indicates that the sample after propensity score matching did not contain observations for the covariate.

Additional File 1: Table S5: Multivariate hazard ratios (with corresponding 95% CIs and p-values) for risk of diabetic retinopathy by each covariate across QOF exposure definitions after 1:1 propensity score matching, including the adjusted study size (n) and C-statistic (also with corresponding 95% CI).

|  | **Exposure Definition** | | | | | | | | | | |
| --- | --- | --- | --- | --- | --- | --- | --- | --- | --- | --- | --- |
|  | **Achieve HbA1c QOF**  **Target** | | |  | **Achieve Blood Pressure QOF Target** | | |  | **Achieve Cholesterol QOF**  **Target** | | |
|  | **Hazard Ratio** | **95% CI** | **p** |  | **Hazard Ratio** | **95% CI** | **p** |  | **Hazard Ratio** | **95% CI** | **p** |
| ***Exposure*** | 0.94 | 0.89-0.99 | 0.0300 |  | 0.87 | 0.83-0.92 | <0.0001 |  | 1.03 | 0.97-1.10 | 0.2918 |
| ***Age*** | 0.99 | 0.99-0.99 | <0.0001 |  | 0.99 | 0.99-0.99 | <0.0001 |  | 0.98 | 0.98-0.99 | <0.0001 |
| ***Sex: Female*** | 1.63 | 1.53-1.73 | <0.0001 |  | 1.63 | 1.54-1.72 | <0.0001 |  | 1.66 | 1.55-1.78 | <0.0001 |
| ***Ethnicity: Asian*** | 0.88 | 0.79-0.99 | 0.0283 |  | 0.91 | 0.82-1.01 | 0.0642 |  | 0.90 | 0.78-1.03 | 0.1115 |
| ***Ethnicity: Black*** | 0.98 | 0.83-1.15 | 0.7927 |  | 0.96 | 0.83-1.12 | 0.6044 |  | 1.05 | 0.88-1.25 | 0.5894 |
| ***Ethnicity: Mixed*** | 1.46 | 1.14-1.87 | 0.0029 |  | 1.40 | 1.13-1.73 | 0.0018 |  | 1.66 | 1.24-2.23 | 0.0007 |
| ***Ethnicity: Other*** | 0.97 | 0.76-1.23 | 0.7835 |  | 0.94 | 0.75-1.17 | 0.5716 |  | 0.82 | 0.62-1.10 | 0.1858 |
| ***IMD*** | 1.00 | 0.99-1.00 | 0.5277 |  | 1.00 | 0.99-1.00 | 0.2198 |  | 0.99 | 0.99-1.00 | 0.0345 |
| ***North West*** | 1.05 | 0.87-1.27 | 0.5970 |  | 1.02 | 0.87-1.20 | 0.8210 |  | 1.02 | 0.83-1.24 | 0.8650 |
| ***Yorkshire & Humber*** | 1.15 | 0.93-1.43 | 0.1954 |  | 1.10 | 0.91-1.33 | 0.3289 |  | 0.91 | 0.71-1.16 | 0.4480 |
| ***East Midlands*** | 0.97 | 0.76-1.25 | 0.8406 |  | 0.94 | 0.75-1.16 | 0.5520 |  | 0.89 | 0.68-1.18 | 0.4269 |
| ***West Midlands*** | 1.05 | 0.86-1.26 | 0.6460 |  | 1.01 | 0.86-1.20 | 0.8601 |  | 0.95 | 0.77-1.16 | 0.5946 |
| ***East of England*** | 0.95 | 0.78-1.16 | 0.6386 |  | 0.95 | 0.81-1.13 | 0.5822 |  | 0.86 | 0.70-1.07 | 0.1808 |
| ***South West*** | 1.04 | 0.86-1.25 | 0.7181 |  | 1.03 | 0.88-1.22 | 0.6841 |  | 0.90 | 0.74-1.11 | 0.3368 |
| ***South Central*** | 1.08 | 0.89-1.31 | 0.4187 |  | 1.00 | 0.85-1.19 | 0.9618 |  | 0.93 | 0.76-1.15 | 0.5138 |
| ***London*** | 1.09 | 0.90-1.31 | 0.3772 |  | 1.03 | 0.87-1.21 | 0.7580 |  | 0.92 | 0.75-1.13 | 0.4274 |
| ***South East Coast*** | 1.03 | 0.85-1.25 | 0.7394 |  | 0.94 | 0.79-1.11 | 0.4358 |  | 0.85 | 0.69-1.05 | 0.1405 |
| ***BMI: Underweight*** | 1.31 | 0.94-1.82 | 0.1116 |  | 1.44 | 1.05-1.96 | 0.0218 |  | 1.36 | 0.93-2.00 | 0.1093 |
| ***BMI: Overweight*** | 1.26 | 0.91-1.75 | 0.1613 |  | 1.39 | 1.03-1.89 | 0.0340 |  | 1.39 | 0.96-2.02 | 0.0847 |
| ***BMI: Obese*** | 1.16 | 0.83-1.60 | 0.3822 |  | 1.27 | 0.94-1.73 | 0.1221 |  | 1.23 | 0.84-1.79 | 0.2816 |
| ***BMI: Missing*** | 0.94 | 0.62-1.44 | 0.7904 |  | 0.97 | 0.66-1.44 | 0.8805 |  | 0.87 | 0.53-1.41 | 0.5725 |
| ***Ex-Smoker*** | 0.92 | 0.87-0.98 | 0.0111 |  | 0.91 | 0.86-0.96 | 0.0007 |  | 0.99 | 0.92-1.06 | 0.7480 |
| ***Current Smoker*** | 0.80 | 0.74-0.87 | <0.0001 |  | 0.81 | 0.75-0.87 | <0.0001 |  | 0.80 | 0.73-0.87 | <0.0001 |
| ***Smoking: Missing*** | 2.84 | 1.95-4.13 | <0.0001 |  | 3.11 | 2.23-4.34 | <0.0001 |  | 2.86 | 1.86-4.40 | <0.0001 |
| ***Alcohol: 1-14*** | 0.89 | 0.83-0.96 | 0.0020 |  | 0.91 | 0.85-0.97 | 0.0041 |  | 0.88 | 0.81-0.96 | 0.0025 |
| ***Alcohol: 15-42*** | 0.97 | 0.87-1.08 | 0.5313 |  | 1.00 | 0.91-1.10 | 0.9561 |  | 1.04 | 0.92-1.18 | 0.5041 |
| ***Alcohol: >42*** | 0.91 | 0.75-1.11 | 0.3534 |  | 0.87 | 0.74-1.03 | 0.1079 |  | 0.80 | 0.64-0.99 | 0.0385 |
| ***Alcohol: Missing*** | 0.82 | 0.75-0.90 | 0.0001 |  | 0.84 | 0.77-0.91 | 0.0001 |  | 0.79 | 0.70-0.88 | <0.0001 |
| ***Morbidities*** | 0.77 | 0.76-0.79 | <0.0001 |  | 0.78 | 0.76-0.79 | <0.0001 |  | 0.79 | 0.77-0.81 | <0.0001 |
| ***Prescriptions*** | 1.00 | 1.00-1.00 | 0.5762 |  | 1.00 | 1.00-1.00 | 0.6968 |  | 1.00 | 0.99-1.00 | 0.8086 |
| ***Hospitalisations*** | 0.92 | 0.87-0.98 | 0.0074 |  | 0.92 | 0.87-0.97 | 0.0013 |  | 0.91 | 0.85-0.97 | 0.0043 |
| ***Duration of diabetes (years)*** | 0.99 | 0.99-1.00 | 0.0396 |  | 1.00 | 0.99-1.00 | 0.1037 |  | 1.00 | 0.99-1.00 | 0.2376 |
| ***Complications*** | 2.10 | 2.05-2.15 | <0.0001 |  | 2.13 | 2.08-2.17 | <0.0001 |  | 2.16 | 2.10-2.22 | <0.0001 |
| ***Glucose lowering therapies*** | 1.09 | 1.06-1.13 | <0.0001 |  | 1.09 | 1.06-1.13 | <0.0001 |  | 1.10 | 1.06-1.15 | <0.0001 |
| ***Insulin prescription*** | 1.04 | 0.96-1.12 | 0.3376 |  | 1.03 | 0.95-1.11 | 0.4774 |  | 1.06 | 0.96-1.16 | 0.2513 |
|  |  |  |  |  |  |  |  |  |  |  |  |
|  | **Value** | **95% CI (lower)** | **95% CI (upper)** |  | **Value** | **95% CI (lower)** | **95% CI (upper)** |  | **Value** | **95% CI (lower)** | **95% CI (upper)** |
| ***n after matching*** | 37,182 |  |  |  | 50,426 |  |  |  | 30,978 |  |  |
| ***C-statistic*** | 0.7498 | 0.7498 | 0.7499 |  | 0.7545 | 0.7545 | 0.7545 |  | 0.7597 | 0.7597 | 0.7598 |

Reference groups for categorical covariates include: white (ethnicity); North East (region); normal weight (BMI); non-smoker (smoking status); 0 units (alcohol consumption); and no insulin prescription (insulin use).

Additional File 1: Table S6: Multivariate hazard ratios (with corresponding 95% CIs and p-values) for risk of diabetic retinopathy by each covariate across NDA exposure definitions after 1:1 propensity score matching, including the adjusted study size (n) and C-statistic (also with corresponding 95% CI).

|  | **Exposure Definition** | | | | | | | | | | |
| --- | --- | --- | --- | --- | --- | --- | --- | --- | --- | --- | --- |
|  | **Meet 4-6 NDA Processes (vs. Meet 0-3 NDA Processes)** | | |  | **Meet 7-9 NDA Processes (vs. Meet 0-3 NDA Processes)** | | |  | **Meet 7-9 NDA Processes (vs. Meet 4-6 NDA Processes)** | | |
|  | **Hazard Ratio** | **95% CI** | **p** |  | **Hazard Ratio** | **95% CI** | **p** |  | **Hazard Ratio** | **95% CI** | **p** |
| ***Exposure*** | 1.16 | 0.98-1.36 | 0.0793 |  | 1.07 | 0.98-1.18 | 0.1378 |  | 1.15 | 0.98-1.35 | 0.0942 |
| ***Age*** | 0.98 | 0.98-0.99 | <0.0001 |  | 0.98 | 0.98-0.99 | <0.0001 |  | 0.98 | 0.98-0.99 | <0.0001 |
| ***Sex: Female*** | 1.55 | 1.29-1.87 | <0.0001 |  | 1.60 | 1.44-1.77 | <0.0001 |  | 1.55 | 1.30-1.85 | <0.0001 |
| ***Ethnicity: Asian*** | 0.87 | 0.63-1.18 | 0.3658 |  | 0.90 | 0.74-1.09 | 0.2724 |  | 0.79 | 0.56-1.11 | 0.1747 |
| ***Ethnicity: Black*** | 0.74 | 0.49-1.13 | 0.1665 |  | 1.08 | 0.85-1.39 | 0.5238 |  | 0.94 | 0.64-1.39 | 0.7542 |
| ***Ethnicity: Mixed*** | 2.74 | 1.56-4.79 | 0.0004 |  | 1.85 | 1.21-2.82 | 0.0046 |  | 1.61 | 0.85-3.03 | 0.1408 |
| ***Ethnicity: Other*** | 0.93 | 0.48-1.82 | 0.8289 |  | 0.88 | 0.57-1.36 | 0.5722 |  | 1.42 | 0.84-2.41 | 0.1919 |
| ***IMD*** | 0.99 | 0.98-1.01 | 0.4091 |  | 1.00 | 0.99-1.01 | 0.5170 |  | 0.99 | 0.97-1.00 | 0.0624 |
| ***North West*** | 1.24 | 0.66-2.32 | 0.5099 |  | 1.03 | 0.75-1.43 | 0.8410 |  | 1.06 | 0.59-1.90 | 0.8519 |
| ***Yorkshire & Humber*** | 2.09 | 1.05-4.16 | 0.0362 |  | 1.12 | 0.77-1.64 | 0.5522 |  | 1.41 | 0.72-2.74 | 0.3148 |
| ***East Midlands*** | 1.11 | 0.51-2.43 | 0.7940 |  | 0.82 | 0.53-1.26 | 0.3653 |  | 0.76 | 0.36-1.60 | 0.4766 |
| ***West Midlands*** | 1.43 | 0.76-2.69 | 0.2713 |  | 1.05 | 0.76-1.47 | 0.7576 |  | 1.20 | 0.66-2.17 | 0.5466 |
| ***East of England*** | 1.32 | 0.69-2.54 | 0.3974 |  | 0.94 | 0.67-1.32 | 0.7137 |  | 1.19 | 0.66-2.15 | 0.5688 |
| ***South West*** | 1.19 | 0.63-2.27 | 0.5871 |  | 0.92 | 0.66-1.29 | 0.6350 |  | 1.10 | 0.61-1.98 | 0.7483 |
| ***South Central*** | 1.56 | 0.83-2.95 | 0.1710 |  | 1.08 | 0.78-1.51 | 0.6322 |  | 1.01 | 0.55-1.84 | 0.9822 |
| ***London*** | 1.27 | 0.67-2.41 | 0.4561 |  | 1.15 | 0.83-1.59 | 0.4130 |  | 1.15 | 0.64-2.08 | 0.6358 |
| ***South East Coast*** | 1.41 | 0.74-2.67 | 0.2982 |  | 0.92 | 0.66-1.28 | 0.6186 |  | 1.16 | 0.64-2.09 | 0.6328 |
| ***BMI: Underweight*** | 0.68 | 0.35-1.29 | 0.2385 |  | 1.62 | 0.91-2.90 | 0.1030 |  | 0.96 | 0.48-1.91 | 0.8986 |
| ***BMI: Overweight*** | 0.69 | 0.37-1.27 | 0.2322 |  | 1.47 | 0.83-2.61 | 0.1895 |  | 0.87 | 0.44-1.71 | 0.6886 |
| ***BMI: Obese*** | 0.58 | 0.31-1.08 | 0.0848 |  | 1.42 | 0.80-2.52 | 0.2299 |  | 0.70 | 0.36-1.37 | 0.2955 |
| ***BMI: Missing*** | 0.46 | 0.19-1.10 | 0.0792 |  | 0.72 | 0.34-1.52 | 0.3906 |  | 0.73 | 0.32-1.65 | 0.4474 |
| ***Ex-Smoker*** | 0.98 | 0.81-1.19 | 0.8756 |  | 0.91 | 0.81-1.01 | 0.0721 |  | 1.04 | 0.86-1.25 | 0.7005 |
| ***Current Smoker*** | 0.69 | 0.55-0.88 | 0.0020 |  | 0.82 | 0.71-0.95 | 0.0088 |  | 0.66 | 0.52-0.83 | 0.0003 |
| ***Smoking: Missing*** | 2.74 | 1.28-5.86 | 0.0091 |  | 2.85 | 1.61-5.05 | 0.0003 |  | 3.98 | 2.14-7.41 | <0.0001 |
| ***Alcohol: 1-14*** | 0.88 | 0.70-1.10 | 0.2518 |  | 0.95 | 0.83-1.08 | 0.4107 |  | 1.04 | 0.83-1.30 | 0.7524 |
| ***Alcohol: 15-42*** | 1.27 | 0.93-1.73 | 0.1307 |  | 1.12 | 0.93-1.35 | 0.2504 |  | 1.05 | 0.76-1.45 | 0.7596 |
| ***Alcohol: >42*** | 0.95 | 0.59-1.54 | 0.8479 |  | 1.05 | 0.78-1.41 | 0.7402 |  | 0.82 | 0.48-1.40 | 0.4566 |
| ***Alcohol: Missing*** | 0.75 | 0.57-0.99 | 0.0410 |  | 0.86 | 0.73-1.01 | 0.0712 |  | 1.04 | 0.79-1.38 | 0.7649 |
| ***Morbidities*** | 0.79 | 0.74-0.84 | <0.0001 |  | 0.78 | 0.75-0.80 | <0.0001 |  | 0.74 | 0.69-0.79 | <0.0001 |
| ***Prescriptions*** | 0.99 | 0.98-1.00 | 0.0962 |  | 1.00 | 0.99-1.00 | 0.3702 |  | 0.99 | 0.98-1.01 | 0.4087 |
| ***Hospitalisations*** | 0.83 | 0.69-0.99 | 0.0402 |  | 0.93 | 0.86-1.02 | 0.1089 |  | 1.01 | 0.87-1.17 | 0.8809 |
| ***Duration of diabetes (years)*** | 1.01 | 1.00-1.03 | 0.0774 |  | 1.00 | 0.99-1.01 | 0.5130 |  | 1.01 | 0.99-1.02 | 0.2864 |
| ***Complications*** | 2.12 | 1.98-2.27 | <0.0001 |  | 2.15 | 2.06-2.24 | <0.0001 |  | 2.09 | 1.96-2.23 | <0.0001 |
| ***Glucose lowering therapies*** | 1.28 | 1.15-1.41 | <0.0001 |  | 1.10 | 1.03-1.16 | 0.0017 |  | 1.17 | 1.07-1.28 | 0.0008 |
| ***Insulin prescription*** | 1.12 | 0.85-1.46 | 0.4244 |  | 1.07 | 0.93-1.23 | 0.3382 |  | 1.17 | 0.91-1.51 | 0.2289 |
|  |  |  |  |  |  |  |  |  |  |  |  |
|  | **Value** | **95% CI (lower)** | **95% CI (upper)** |  | **Value** | **95% CI (lower)** | **95% CI (upper)** |  | **Value** | **95% CI (lower)** | **95% CI (upper)** |
| ***n after matching*** | 5,438 |  |  |  | 14,052 |  |  |  | 5,484 |  |  |
| ***C-statistic*** | 0.7744 | 0.7742 | 0.7745 |  | 0.7548 | 0.7547 | 0.7548 |  | 0.7785 | 0.7783 | 0.7786 |

Reference groups for categorical covariates include: white (ethnicity); North East (region); normal weight (BMI); non-smoker (smoking status); 0 units (alcohol consumption); and no insulin prescription (insulin use).

Additional File 1: Table S7: Multivariate hazard ratios (with corresponding 95% CIs and p-values) for risk of diabetic retinopathy by each covariate across QOF and NDA exposure definitions after 1:1 propensity score matching, including the adjusted study size (n), and C-statistic (also with corresponding 95% CI).

|  | **Exposure Definition** | | | | | | | | | | |
| --- | --- | --- | --- | --- | --- | --- | --- | --- | --- | --- | --- |
|  | **Achieve All QOF Targets** | | |  | **Meet All NDA Processes** | | |  | **Achieve All QOF & NDA Targets** | | |
|  | **Hazard Ratio** | **95% CI** | **p** |  | **Hazard Ratio** | **95% CI** | **p** |  | **Hazard Ratio** | **95% CI** | **p** |
| ***Exposure*** | 0.86 | 0.81-0.91 | <0.0001 |  | 1.03 | 0.99-1.08 | 0.1703 |  | 0.92 | 0.85-0.99 | 0.0241 |
| ***Age*** | 0.99 | 0.99-0.99 | <0.0001 |  | 0.99 | 0.98-0.99 | <0.0001 |  | 0.99 | 0.99-1.00 | 0.0005 |
| ***Sex: Female*** | 1.68 | 1.58-1.79 | <0.0001 |  | 1.70 | 1.61-1.79 | <0.0001 |  | 1.64 | 1.51-1.79 | <0.0001 |
| ***Ethnicity: Asian*** | 0.88 | 0.78-1.00 | 0.0560 |  | 0.89 | 0.80-0.99 | 0.0252 |  | 0.81 | 0.66-0.98 | 0.0297 |
| ***Ethnicity: Black*** | 0.99 | 0.80-1.21 | 0.8908 |  | 0.94 | 0.78-1.12 | 0.4671 |  | 0.87 | 0.61-1.23 | 0.4324 |
| ***Ethnicity: Mixed*** | 1.42 | 1.05-1.92 | 0.0224 |  | 1.32 | 1.05-1.65 | 0.0175 |  | 1.39 | 0.93-2.09 | 0.1071 |
| ***Ethnicity: Other*** | 1.00 | 0.76-1.32 | 0.9948 |  | 0.84 | 0.66-1.08 | 0.1784 |  | 1.18 | 0.81-1.71 | 0.3835 |
| ***IMD*** | 1.00 | 0.99-1.00 | 0.4650 |  | 1.00 | 0.99-1.00 | 0.4123 |  | 1.00 | 0.99-1.00 | 0.2749 |
| ***North West*** | 1.02 | 0.85-1.22 | 0.8359 |  | 1.04 | 0.89-1.22 | 0.6183 |  | 0.96 | 0.74-1.23 | 0.7329 |
| ***Yorkshire & Humber*** | 1.04 | 0.83-1.29 | 0.7351 |  | 1.08 | 0.89-1.30 | 0.4439 |  | 0.90 | 0.66-1.22 | 0.4929 |
| ***East Midlands*** | 0.95 | 0.74-1.22 | 0.6917 |  | 0.97 | 0.78-1.20 | 0.7634 |  | 0.75 | 0.52-1.08 | 0.1250 |
| ***West Midlands*** | 1.02 | 0.84-1.23 | 0.8630 |  | 0.99 | 0.84-1.17 | 0.9352 |  | 0.98 | 0.75-1.26 | 0.8518 |
| ***East of England*** | 0.99 | 0.82-1.20 | 0.9112 |  | 0.98 | 0.83-1.16 | 0.8535 |  | 0.89 | 0.69-1.17 | 0.4132 |
| ***South West*** | 0.99 | 0.82-1.20 | 0.9413 |  | 0.99 | 0.84-1.17 | 0.9328 |  | 0.96 | 0.75-1.25 | 0.7811 |
| ***South Central*** | 1.09 | 0.90-1.32 | 0.3726 |  | 1.03 | 0.88-1.22 | 0.7055 |  | 0.96 | 0.74-1.25 | 0.7575 |
| ***London*** | 1.02 | 0.84-1.22 | 0.8723 |  | 1.05 | 0.89-1.23 | 0.5893 |  | 0.97 | 0.75-1.26 | 0.8439 |
| ***South East Coast*** | 0.90 | 0.74-1.09 | 0.2845 |  | 0.95 | 0.81-1.12 | 0.5768 |  | 0.87 | 0.67-1.13 | 0.3033 |
| ***BMI: Underweight*** | 1.62 | 1.13-2.31 | 0.0087 |  | 1.45 | 1.08-1.94 | 0.0134 |  | 1.43 | 0.91-2.24 | 0.1187 |
| ***BMI: Overweight*** | 1.55 | 1.08-2.21 | 0.0163 |  | 1.40 | 1.05-1.87 | 0.0218 |  | 1.35 | 0.87-2.10 | 0.1851 |
| ***BMI: Obese*** | 1.40 | 0.98-2.00 | 0.0631 |  | 1.29 | 0.96-1.72 | 0.0880 |  | 1.28 | 0.82-2.00 | 0.2716 |
| ***BMI: Missing*** | 1.08 | 0.65-1.80 | 0.7567 |  | 1.00 | 0.67-1.51 | 0.9917 |  | 0.71 | 0.32-1.56 | 0.3920 |
| ***Ex-Smoker*** | 0.93 | 0.87-0.99 | 0.0160 |  | 0.91 | 0.86-0.96 | 0.0005 |  | 0.94 | 0.86-1.03 | 0.1652 |
| ***Current Smoker*** | 0.84 | 0.77-0.91 | 0.0001 |  | 0.84 | 0.79-0.91 | <0.0001 |  | 0.90 | 0.80-1.02 | 0.0911 |
| ***Smoking: Missing*** | 3.79 | 2.15-6.70 | <0.0001 |  | N/A | N/A | N/A |  | N/A | N/A | N/A |
| ***Alcohol: 1-14*** | 0.92 | 0.86-0.99 | 0.0309 |  | 0.89 | 0.84-0.95 | 0.0004 |  | 0.94 | 0.85-1.04 | 0.2313 |
| ***Alcohol: 15-42*** | 0.98 | 0.88-1.10 | 0.7411 |  | 0.96 | 0.87-1.06 | 0.3975 |  | 0.81 | 0.69-0.95 | 0.0106 |
| ***Alcohol: >42*** | 0.92 | 0.75-1.13 | 0.4328 |  | 0.84 | 0.70-1.00 | 0.0486 |  | 0.83 | 0.62-1.12 | 0.2274 |
| ***Alcohol: Missing*** | 0.80 | 0.72-0.89 | <0.0001 |  | 0.80 | 0.73-0.87 | <0.0001 |  | 0.84 | 0.71-0.98 | 0.0249 |
| ***Morbidities*** | 0.77 | 0.76-0.79 | <0.0001 |  | 0.77 | 0.75-0.78 | <0.0001 |  | 0.74 | 0.72-0.76 | <0.0001 |
| ***Prescriptions*** | 1.00 | 0.99-1.00 | 0.7650 |  | 1.00 | 1.00-1.00 | 0.9327 |  | 1.01 | 1.00-1.01 | 0.0163 |
| ***Hospitalisations*** | 0.90 | 0.84-0.96 | 0.0010 |  | 0.93 | 0.87-0.98 | 0.0142 |  | 0.90 | 0.81-0.99 | 0.0336 |
| ***Duration of diabetes (years)*** | 0.99 | 0.99-1.00 | 0.0230 |  | 0.99 | 0.99-1.00 | 0.0231 |  | 0.99 | 0.99-1.00 | 0.0525 |
| ***Complications*** | 2.14 | 2.09-2.19 | <0.0001 |  | 2.11 | 2.07-2.16 | <0.0001 |  | 2.17 | 2.10-2.24 | <0.0001 |
| ***Glucose lowering therapies*** | 1.10 | 1.06-1.14 | <0.0001 |  | 1.09 | 1.06-1.13 | <0.0001 |  | 1.06 | 1.01-1.12 | 0.0215 |
| ***Insulin prescription*** | 1.11 | 1.00-1.23 | 0.0440 |  | 1.00 | 0.93-1.08 | 0.9421 |  | 1.03 | 0.89-1.20 | 0.6613 |
|  |  |  |  |  |  |  |  |  |  |  |  |
|  | **Value** | **95% CI (lower)** | **95% CI (upper)** |  | **Value** | **95% CI (lower)** | **95% CI (upper)** |  | **Value** | **95% CI (lower)** | **95% CI (upper)** |
| ***n after matching*** | 40,220 |  |  |  | 49,602 |  |  |  | 19,944 |  |  |
| ***C-statistic*** | 0.7536 | 0.7536 | 0.7536 |  | 0.7478 | 0.7478 | 0.7478 |  | 0.7516 | 0.7516 | 0.7517 |

Reference groups for categorical covariates include: white (ethnicity); North East (region); normal weight (BMI); non-smoker (smoking status); 0 units (alcohol consumption); and no insulin prescription (insulin use).

N/A indicates that the sample after propensity score matching did not contain observations for the covariate.

Additional File 1: Table S8: Univariate hazard ratios (with corresponding 95% CIs and p-values) for risk of diabetic retinopathy by each covariate across QOF exposure definitions, *among those who meet all other QOF targets*, after 1:1 propensity score matching.

|  | **Exposure Definition** | | | | | | | | | | |
| --- | --- | --- | --- | --- | --- | --- | --- | --- | --- | --- | --- |
|  | **Achieve HBA1c QOF**  **Target** | | |  | **Achieve Blood Pressure QOF Target** | | |  | **Achieve Cholesterol QOF**  **Target** | | |
|  | **Hazard Ratio** | **95% CI** | **p** |  | **Hazard Ratio** | **95% CI** | **p** |  | **Hazard Ratio** | **95% CI** | **p** |
| ***Exposure*** | 0.80 | 0.73-0.87 | <0.0001 |  | 0.84 | 0.78-0.90 | <0.0001 |  | 1.05 | 0.93-1.19 | 0.3985 |
| ***Age*** | 1.00 | 1.00-1.00 | 0.6054 |  | 1.01 | 1.00-1.01 | 0.0012 |  | 1.00 | 1.00-1.01 | 0.3069 |
| ***Sex: Female*** | 0.95 | 0.87-1.04 | 0.2749 |  | 0.96 | 0.90-1.03 | 0.2922 |  | 0.93 | 0.83-1.05 | 0.2476 |
| ***Ethnicity: Asian*** | 0.88 | 0.74-1.03 | 0.1083 |  | 0.92 | 0.79-1.07 | 0.2620 |  | 1.09 | 0.83-1.41 | 0.5410 |
| ***Ethnicity: Black*** | 1.11 | 0.82-1.49 | 0.5008 |  | 0.88 | 0.68-1.14 | 0.3349 |  | 0.90 | 0.57-1.42 | 0.6604 |
| ***Ethnicity: Mixed*** | 1.09 | 0.65-1.80 | 0.7502 |  | 1.29 | 0.89-1.86 | 0.1724 |  | 0.86 | 0.38-1.91 | 0.7072 |
| ***Ethnicity: Other*** | 0.96 | 0.64-1.44 | 0.8542 |  | 0.93 | 0.67-1.31 | 0.6919 |  | 1.07 | 0.64-1.77 | 0.8057 |
| ***IMD*** | 1.00 | 0.99-1.00 | 0.3844 |  | 1.00 | 0.99-1.00 | 0.2406 |  | 1.01 | 1.00-1.02 | 0.1788 |
| ***North West*** | 0.88 | 0.79-0.99 | 0.0377 |  | 0.99 | 0.91-1.09 | 0.8909 |  | 1.04 | 0.89-1.22 | 0.5958 |
| ***Yorkshire & Humber*** | 1.03 | 0.83-1.29 | 0.7845 |  | 1.01 | 0.84-1.22 | 0.8777 |  | 0.93 | 0.67-1.29 | 0.6736 |
| ***East Midlands*** | 1.10 | 0.83-1.45 | 0.5116 |  | 1.05 | 0.83-1.33 | 0.6735 |  | 1.26 | 0.87-1.82 | 0.2229 |
| ***West Midlands*** | 0.93 | 0.81-1.07 | 0.3062 |  | 0.97 | 0.87-1.08 | 0.6049 |  | 0.90 | 0.74-1.09 | 0.2761 |
| ***East of England*** | 1.06 | 0.93-1.22 | 0.3822 |  | 0.91 | 0.80-1.02 | 0.1111 |  | 0.92 | 0.75-1.13 | 0.4312 |
| ***South West*** | 0.96 | 0.85-1.09 | 0.5438 |  | 1.10 | 1.00-1.22 | 0.0493 |  | 1.02 | 0.86-1.22 | 0.7836 |
| ***South Central*** | 1.15 | 1.01-1.30 | 0.0316 |  | 1.00 | 0.90-1.12 | 0.9631 |  | 1.11 | 0.93-1.32 | 0.2621 |
| ***London*** | 1.10 | 0.97-1.25 | 0.1316 |  | 1.07 | 0.96-1.18 | 0.2286 |  | 1.09 | 0.92-1.30 | 0.3068 |
| ***South East Coast*** | 0.96 | 0.84-1.10 | 0.5360 |  | 0.93 | 0.83-1.04 | 0.1950 |  | 0.82 | 0.68-1.00 | 0.0488 |
| ***BMI: Underweight*** | 0.99 | 0.87-1.13 | 0.9054 |  | 1.06 | 0.96-1.17 | 0.2256 |  | 1.06 | 0.90-1.24 | 0.4942 |
| ***BMI: Overweight*** | 1.08 | 0.98-1.18 | 0.1039 |  | 1.04 | 0.97-1.12 | 0.3003 |  | 1.09 | 0.96-1.23 | 0.1774 |
| ***BMI: Obese*** | 0.96 | 0.88-1.05 | 0.4046 |  | 0.95 | 0.89-1.02 | 0.1764 |  | 0.91 | 0.80-1.02 | 0.1176 |
| ***BMI: Missing*** | 0.55 | 0.30-0.99 | 0.0458 |  | 0.76 | 0.51-1.13 | 0.1787 |  | 0.57 | 0.24-1.37 | 0.2078 |
| ***Ex-Smoker*** | 1.04 | 0.95-1.14 | 0.3591 |  | 1.00 | 0.93-1.07 | 0.9982 |  | 1.05 | 0.93-1.19 | 0.4226 |
| ***Current Smoker*** | 1.00 | 0.89-1.14 | 0.9540 |  | 0.96 | 0.86-1.07 | 0.4166 |  | 0.92 | 0.78-1.10 | 0.3823 |
| ***Smoking: Missing*** | 3.00 | 1.66-5.43 | 0.0003 |  | 2.49 | 1.38-4.50 | 0.0025 |  | 2.60 | 0.65-10.39 | 0.1778 |
| ***Alcohol: 1-14*** | 0.99 | 0.91-1.09 | 0.8961 |  | 0.97 | 0.91-1.05 | 0.4631 |  | 1.09 | 0.97-1.24 | 0.1578 |
| ***Alcohol: 15-42*** | 0.92 | 0.78-1.08 | 0.3117 |  | 0.99 | 0.88-1.11 | 0.8537 |  | 0.96 | 0.78-1.18 | 0.6835 |
| ***Alcohol: >42*** | 0.82 | 0.57-1.17 | 0.2782 |  | 0.86 | 0.66-1.12 | 0.2747 |  | 0.82 | 0.51-1.30 | 0.3917 |
| ***Alcohol: Missing*** | 0.84 | 0.74-0.96 | 0.0096 |  | 0.83 | 0.74-0.93 | 0.0017 |  | 0.84 | 0.69-1.03 | 0.0917 |
| ***Morbidities*** | 0.99 | 0.96-1.01 | 0.3463 |  | 1.02 | 1.00-1.05 | 0.0371 |  | 0.98 | 0.95-1.02 | 0.3740 |
| ***Prescriptions*** | 1.01 | 1.00-1.01 | 0.0005 |  | 1.01 | 1.00-1.01 | 0.0017 |  | 1.01 | 1.01-1.02 | 0.0005 |
| ***Hospitalisations*** | 1.10 | 1.02-1.19 | 0.0195 |  | 1.04 | 0.96-1.12 | 0.3647 |  | 1.03 | 0.91-1.15 | 0.6702 |
| ***Duration of diabetes (years)*** | 1.00 | 0.99-1.01 | 0.9399 |  | 1.00 | 1.00-1.01 | 0.5536 |  | 1.01 | 1.00-1.02 | 0.1642 |
| ***Complications*** | 1.68 | 1.63-1.74 | <0.0001 |  | 1.75 | 1.71-1.80 | <0.0001 |  | 1.77 | 1.70-1.85 | <0.0001 |
| ***Glucose lowering therapies*** | 1.19 | 1.14-1.24 | <0.0001 |  | 1.17 | 1.13-1.22 | <0.0001 |  | 1.26 | 1.18-1.34 | <0.0001 |
| ***Insulin prescription*** | 1.47 | 1.33-1.63 | <0.0001 |  | 1.55 | 1.36-1.76 | <0.0001 |  | 1.54 | 1.24-1.91 | 0.0001 |

Study sizes across exposures after 1:1 propensity score matching are found in Supplementary Table 8, as they are the same between univariate and multivariate analyses.

Additional File 1: Table S9: Multivariate hazard ratios (with corresponding 95% CIs and p-values) for risk of diabetic retinopathy by each covariate across QOF exposure definitions, *among those who meet all other QOF targets*, after 1:1 propensity score matching, including the adjusted study size (n) and C-statistic (also with corresponding 95% CI).

|  | **Exposure Definition** | | | | | | | | | | |
| --- | --- | --- | --- | --- | --- | --- | --- | --- | --- | --- | --- |
|  | **Achieve HbA1c QOF Target** | | |  | **Achieve Blood Pressure QOF Target** | | |  | **Achieve Cholesterol QOF Target** | | |
|  | **Hazard Ratio** | **95% CI** | **p** |  | **Hazard Ratio** | **95% CI** | **p** |  | **Hazard Ratio** | **95% CI** | **p** |
| ***Exposure*** | 0.85 | 0.78-0.92 | 0.0002 |  | 0.81 | 0.76-0.87 | <0.0001 |  | 1.00 | 0.89-1.13 | 0.9662 |
| ***Age*** | 0.99 | 0.99-1.00 | <0.0001 |  | 0.99 | 0.99-0.99 | <0.0001 |  | 0.99 | 0.98-1.00 | 0.0004 |
| ***Sex: Female*** | 1.68 | 1.52-1.85 | <0.0001 |  | 1.63 | 1.50-1.76 | <0.0001 |  | 1.69 | 1.48-1.94 | <0.0001 |
| ***Ethnicity: Asian*** | 0.83 | 0.69-0.99 | 0.0378 |  | 0.87 | 0.74-1.02 | 0.0837 |  | 1.05 | 0.79-1.39 | 0.7423 |
| ***Ethnicity: Black*** | 1.17 | 0.87-1.58 | 0.2985 |  | 0.89 | 0.69-1.15 | 0.3748 |  | 0.97 | 0.61-1.53 | 0.8918 |
| ***Ethnicity: Mixed*** | 0.99 | 0.59-1.65 | 0.9668 |  | 1.02 | 0.70-1.47 | 0.9220 |  | 0.84 | 0.37-1.88 | 0.6682 |
| ***Ethnicity: Other*** | 1.11 | 0.74-1.68 | 0.6025 |  | 0.95 | 0.68-1.34 | 0.7679 |  | 1.07 | 0.64-1.81 | 0.7855 |
| ***IMD*** | 1.00 | 0.99-1.00 | 0.3936 |  | 1.00 | 0.99-1.00 | 0.1810 |  | 1.00 | 0.99-1.02 | 0.4608 |
| ***North West*** | 1.05 | 0.77-1.43 | 0.7678 |  | 1.04 | 0.82-1.32 | 0.7651 |  | 0.89 | 0.60-1.31 | 0.5618 |
| ***Yorkshire & Humber*** | 1.19 | 0.83-1.72 | 0.3428 |  | 1.01 | 0.76-1.35 | 0.9556 |  | 0.81 | 0.50-1.32 | 0.3956 |
| ***East Midlands*** | 1.15 | 0.77-1.72 | 0.4946 |  | 1.08 | 0.78-1.50 | 0.6238 |  | 1.18 | 0.71-1.98 | 0.5183 |
| ***West Midlands*** | 1.00 | 0.73-1.38 | 0.9828 |  | 1.00 | 0.78-1.28 | 0.9983 |  | 0.77 | 0.52-1.16 | 0.2184 |
| ***East of England*** | 1.16 | 0.84-1.60 | 0.3653 |  | 0.93 | 0.72-1.20 | 0.5681 |  | 0.90 | 0.59-1.37 | 0.6177 |
| ***South West*** | 1.06 | 0.77-1.45 | 0.7173 |  | 1.06 | 0.83-1.36 | 0.6321 |  | 0.85 | 0.57-1.26 | 0.4133 |
| ***South Central*** | 1.23 | 0.90-1.69 | 0.1969 |  | 1.01 | 0.79-1.30 | 0.9087 |  | 0.95 | 0.63-1.42 | 0.8041 |
| ***London*** | 1.25 | 0.91-1.70 | 0.1663 |  | 1.08 | 0.84-1.37 | 0.5623 |  | 0.92 | 0.62-1.37 | 0.6915 |
| ***South East Coast*** | 1.07 | 0.78-1.47 | 0.6711 |  | 0.94 | 0.73-1.21 | 0.6402 |  | 0.69 | 0.46-1.04 | 0.0763 |
| ***BMI: Underweight*** | 1.80 | 0.96-3.40 | 0.0672 |  | 1.60 | 1.01-2.54 | 0.0449 |  | 1.60 | 0.75-3.42 | 0.2217 |
| ***BMI: Overweight*** | 1.77 | 0.95-3.31 | 0.0736 |  | 1.51 | 0.95-2.37 | 0.0781 |  | 1.51 | 0.71-3.21 | 0.2794 |
| ***BMI: Obese*** | 1.57 | 0.84-2.93 | 0.1566 |  | 1.40 | 0.89-2.20 | 0.1500 |  | 1.43 | 0.68-3.04 | 0.3469 |
| ***BMI: Missing*** | 1.25 | 0.53-2.95 | 0.6122 |  | 1.17 | 0.64-2.12 | 0.6129 |  | 1.07 | 0.34-3.40 | 0.9032 |
| ***Ex-Smoker*** | 0.96 | 0.87-1.06 | 0.4036 |  | 0.88 | 0.81-0.95 | 0.0012 |  | 0.89 | 0.77-1.02 | 0.1012 |
| ***Current Smoker*** | 0.85 | 0.74-0.98 | 0.0211 |  | 0.86 | 0.76-0.96 | 0.0098 |  | 0.87 | 0.72-1.05 | 0.1507 |
| ***Smoking: Missing*** | 4.64 | 2.55-8.44 | <0.0001 |  | 2.90 | 1.60-5.26 | 0.0004 |  | 6.07 | 1.50-24.62 | 0.0116 |
| ***Alcohol: 1-14*** | 0.90 | 0.80-1.01 | 0.0690 |  | 0.88 | 0.80-0.97 | 0.0097 |  | 1.10 | 0.92-1.30 | 0.2975 |
| ***Alcohol: 15-42*** | 0.84 | 0.69-1.01 | 0.0657 |  | 0.89 | 0.77-1.03 | 0.1207 |  | 1.03 | 0.79-1.33 | 0.8360 |
| ***Alcohol: >42*** | 0.71 | 0.49-1.03 | 0.0700 |  | 0.70 | 0.53-0.93 | 0.0126 |  | 0.86 | 0.52-1.41 | 0.5492 |
| ***Alcohol: Missing*** | 0.77 | 0.65-0.90 | 0.0013 |  | 0.77 | 0.67-0.88 | 0.0002 |  | 0.92 | 0.72-1.17 | 0.5105 |
| ***Morbidities*** | 0.75 | 0.73-0.78 | <0.0001 |  | 0.77 | 0.75-0.79 | <0.0001 |  | 0.75 | 0.72-0.79 | <0.0001 |
| ***Prescriptions*** | 1.00 | 1.00-1.01 | 0.7792 |  | 1.00 | 0.99-1.01 | 0.8361 |  | 1.00 | 0.99-1.01 | 0.8241 |
| ***Hospitalisations*** | 0.99 | 0.91-1.08 | 0.8765 |  | 0.93 | 0.86-1.01 | 0.0980 |  | 0.99 | 0.87-1.12 | 0.8303 |
| ***Duration of diabetes (years)*** | 0.99 | 0.98-1.00 | 0.0532 |  | 1.00 | 0.99-1.00 | 0.2497 |  | 1.00 | 0.99-1.02 | 0.4251 |
| ***Complications*** | 2.10 | 2.02-2.18 | <0.0001 |  | 2.19 | 2.13-2.26 | <0.0001 |  | 2.22 | 2.10-2.34 | <0.0001 |
| ***Glucose lowering therapies*** | 1.12 | 1.06-1.18 | 0.0001 |  | 1.07 | 1.02-1.12 | 0.0074 |  | 1.09 | 1.01-1.19 | 0.0343 |
| ***Insulin prescription*** | 1.08 | 0.97-1.21 | 0.1566 |  | 1.01 | 0.87-1.16 | 0.9257 |  | 1.04 | 0.82-1.32 | 0.7719 |
|  |  |  |  |  |  |  |  |  |  |  |  |
|  | **Value** | **95% CI (lower)** | **95% CI (upper)** |  | **Value** | **95% CI (lower)** | **95% CI (upper)** |  | **Value** | **95% CI (lower)** | **95% CI (upper)** |
| ***n after matching*** | 13,736 |  |  |  | 23,640 |  |  |  | 9,072 |  |  |
| ***C-statistic*** | 0.7498 | 0.7497 | 0.7498 |  | 0.7557 | 0.7556 | 0.7557 |  | 0.7660 | 0.7659 | 0.7661 |

Reference groups for categorical covariates include: white (ethnicity); North East (region); normal weight (BMI); non-smoker (smoking status); 0 units (alcohol consumption); and no insulin prescription (insulin use).

Additional File 1: Table S10: Univariate hazard ratios (with corresponding 95% CIs and p-values) for risk of sight-threatening diabetic retinopathy by each covariate across QOF exposure definitions after 1:1 propensity score matching.

|  | **Exposure Definition** | | | | | | | | | | |
| --- | --- | --- | --- | --- | --- | --- | --- | --- | --- | --- | --- |
|  | **Achieve HbA1c QOF**  **Target** | | |  | **Achieve Blood Pressure QOF Target** | | |  | **Achieve Cholesterol QOF**  **Target** | | |
|  | **Hazard Ratio** | **95% CI** | **p** |  | **Hazard Ratio** | **95% CI** | **p** |  | **Hazard Ratio** | **95% CI** | **p** |
| ***Exposure*** | 0.66 | 0.56-0.78 | <0.0001 |  | 0.79 | 0.68-0.92 | 0.0022 |  | 0.80 | 0.66-0.97 | 0.0249 |
| ***Age*** | 0.99 | 0.98-0.99 | <0.0001 |  | 0.98 | 0.98-0.99 | <0.0001 |  | 0.98 | 0.97-0.99 | <0.0001 |
| ***Sex: Female*** | 0.91 | 0.77-1.07 | 0.2352 |  | 0.86 | 0.74-1.00 | 0.0437 |  | 0.92 | 0.76-1.12 | 0.4186 |
| ***Ethnicity: Asian*** | 1.42 | 1.09-1.84 | 0.0082 |  | 1.40 | 1.08-1.81 | 0.0098 |  | 1.22 | 0.85-1.75 | 0.2808 |
| ***Ethnicity: Black*** | 1.47 | 0.98-2.19 | 0.0605 |  | 1.58 | 1.09-2.29 | 0.0157 |  | 2.16 | 1.44-3.23 | 0.0002 |
| ***Ethnicity: Mixed*** | 1.36 | 0.61-3.03 | 0.4580 |  | 1.77 | 0.95-3.30 | 0.0739 |  | 2.09 | 0.93-4.67 | 0.0737 |
| ***Ethnicity: Other*** | 1.02 | 0.51-2.05 | 0.9594 |  | 1.12 | 0.60-2.10 | 0.7148 |  | 1.02 | 0.46-2.29 | 0.9529 |
| ***IMD*** | 1.00 | 0.98-1.01 | 0.8317 |  | 0.99 | 0.98-1.01 | 0.3322 |  | 0.99 | 0.97-1.01 | 0.3621 |
| ***North West*** | 1.08 | 0.88-1.32 | 0.4834 |  | 1.05 | 0.87-1.27 | 0.5995 |  | 1.10 | 0.86-1.41 | 0.4265 |
| ***Yorkshire & Humber*** | 0.90 | 0.58-1.39 | 0.6346 |  | 0.87 | 0.58-1.30 | 0.4917 |  | 0.80 | 0.45-1.42 | 0.4402 |
| ***East Midlands*** | 0.91 | 0.51-1.60 | 0.7355 |  | 0.98 | 0.58-1.63 | 0.9230 |  | 0.85 | 0.42-1.71 | 0.6490 |
| ***West Midlands*** | 0.90 | 0.70-1.17 | 0.4350 |  | 0.96 | 0.76-1.21 | 0.7304 |  | 1.05 | 0.79-1.41 | 0.7267 |
| ***East of England*** | 1.17 | 0.91-1.50 | 0.2112 |  | 1.08 | 0.86-1.37 | 0.5118 |  | 0.94 | 0.68-1.31 | 0.7192 |
| ***South West*** | 0.99 | 0.78-1.25 | 0.9239 |  | 1.18 | 0.96-1.44 | 0.1177 |  | 1.01 | 0.77-1.35 | 0.9188 |
| ***South Central*** | 0.86 | 0.67-1.12 | 0.2638 |  | 0.78 | 0.61-1.00 | 0.0506 |  | 0.91 | 0.67-1.24 | 0.5613 |
| ***London*** | 1.01 | 0.80-1.28 | 0.9228 |  | 1.01 | 0.81-1.25 | 0.9456 |  | 0.99 | 0.75-1.32 | 0.9486 |
| ***South East Coast*** | 1.13 | 0.90-1.42 | 0.2770 |  | 1.08 | 0.87-1.34 | 0.4614 |  | 1.12 | 0.85-1.47 | 0.4299 |
| ***BMI: Underweight*** | 1.14 | 0.90-1.44 | 0.2759 |  | 1.12 | 0.90-1.38 | 0.3028 |  | 0.93 | 0.70-1.25 | 0.6396 |
| ***BMI: Overweight*** | 0.93 | 0.78-1.11 | 0.4034 |  | 0.96 | 0.82-1.13 | 0.6492 |  | 0.99 | 0.80-1.22 | 0.9171 |
| ***BMI: Obese*** | 1.02 | 0.87-1.20 | 0.8102 |  | 1.00 | 0.86-1.16 | 0.9935 |  | 1.05 | 0.86-1.27 | 0.6238 |
| ***BMI: Missing*** | 0.73 | 0.33-1.64 | 0.4489 |  | 0.69 | 0.31-1.53 | 0.3602 |  | 0.86 | 0.36-2.09 | 0.7463 |
| ***Ex-Smoker*** | 0.93 | 0.79-1.10 | 0.4109 |  | 0.93 | 0.80-1.09 | 0.3689 |  | 0.88 | 0.71-1.09 | 0.2327 |
| ***Current Smoker*** | 0.88 | 0.70-1.11 | 0.2824 |  | 0.95 | 0.77-1.17 | 0.6266 |  | 0.93 | 0.72-1.22 | 0.6157 |
| ***Smoking: Missing*** | 0.62 | 0.09-4.44 | 0.6371 |  | 1.35 | 0.34-5.42 | 0.6690 |  | 1.91 | 0.48-7.67 | 0.3608 |
| ***Alcohol: 1-14*** | 0.93 | 0.79-1.09 | 0.3578 |  | 0.93 | 0.80-1.08 | 0.3570 |  | 0.86 | 0.71-1.04 | 0.1220 |
| ***Alcohol: 15-42*** | 0.79 | 0.58-1.06 | 0.1162 |  | 0.71 | 0.54-0.94 | 0.0172 |  | 0.67 | 0.46-0.98 | 0.0399 |
| ***Alcohol: >42*** | 0.93 | 0.53-1.65 | 0.8108 |  | 0.96 | 0.58-1.57 | 0.8577 |  | 0.93 | 0.50-1.74 | 0.8239 |
| ***Alcohol: Missing*** | 1.26 | 1.02-1.56 | 0.0309 |  | 1.27 | 1.04-1.55 | 0.0198 |  | 1.37 | 1.07-1.76 | 0.0133 |
| ***Morbidities*** | 0.94 | 0.89-0.99 | 0.0236 |  | 0.96 | 0.92-1.01 | 0.1341 |  | 0.98 | 0.92-1.04 | 0.4620 |
| ***Prescriptions*** | 1.01 | 1.01-1.02 | 0.0001 |  | 1.02 | 1.02-1.03 | <0.0001 |  | 1.02 | 1.01-1.03 | <0.0001 |
| ***Hospitalisations*** | 1.04 | 0.89-1.23 | 0.6118 |  | 1.01 | 0.86-1.19 | 0.9127 |  | 1.08 | 0.90-1.29 | 0.4221 |
| ***Duration of diabetes (years)*** | 1.00 | 0.99-1.02 | 0.7312 |  | 1.01 | 1.00-1.02 | 0.0835 |  | 1.01 | 1.00-1.03 | 0.1172 |
| ***Complications*** | 1.62 | 1.53-1.72 | <0.0001 |  | 1.71 | 1.62-1.80 | <0.0001 |  | 1.75 | 1.63-1.87 | <0.0001 |
| ***Glucose lowering therapies*** | 1.45 | 1.34-1.56 | <0.0001 |  | 1.57 | 1.47-1.68 | <0.0001 |  | 1.60 | 1.47-1.74 | <0.0001 |
| ***Insulin prescription*** | 2.10 | 1.74-2.53 | <0.0001 |  | 2.59 | 2.17-3.09 | <0.0001 |  | 2.93 | 2.34-3.67 | <0.0001 |

Study sizes across exposures after 1:1 propensity score matching are found in Supplementary Table 4, as they are the same between univariate and multivariate analyses.

Additional File 1: Table S11: Univariate hazard ratios (with corresponding 95% CIs and p-values) for risk of sight-threatening diabetic retinopathy by each covariate across NDA exposure definitions after 1:1 propensity score matching.

|  | **Exposure Definition** | | | | | | | | | | |
| --- | --- | --- | --- | --- | --- | --- | --- | --- | --- | --- | --- |
|  | **Meet 4-6 NDA Processes (vs. Meet 0-3 NDA Processes)** | | |  | **Meet 7-9 NDA Processes (vs. Meet 0-3 NDA Processes)** | | |  | **Meet 7-9 NDA Processes (vs. Meet 4-6 NDA Processes)** | | |
|  | **Hazard Ratio** | **95% CI** | **p** |  | **Hazard Ratio** | **95% CI** | **p** |  | **Hazard Ratio** | **95% CI** | **p** |
| ***Exposure*** | 1.10 | 0.71-1.69 | 0.6834 |  | 0.75 | 0.57-0.98 | 0.0325 |  | 1.07 | 0.70-1.63 | 0.7669 |
| ***Age*** | 0.98 | 0.97-1.00 | 0.0167 |  | 0.98 | 0.97-0.99 | 0.0001 |  | 0.98 | 0.96-0.99 | 0.0041 |
| ***Sex: Female*** | 0.79 | 0.50-1.23 | 0.2978 |  | 0.91 | 0.69-1.18 | 0.4735 |  | 0.72 | 0.46-1.12 | 0.1454 |
| ***Ethnicity: Asian*** | 1.12 | 0.51-2.42 | 0.7810 |  | 1.21 | 0.77-1.92 | 0.4077 |  | 0.68 | 0.25-1.87 | 0.4591 |
| ***Ethnicity: Black*** | 2.05 | 0.94-4.45 | 0.0697 |  | 2.59 | 1.62-4.15 | 0.0001 |  | 2.57 | 1.24-5.33 | 0.0110 |
| ***Ethnicity: Mixed*** | 3.16 | 0.78-12.86 | 0.1083 |  | 0.68 | 0.10-4.88 | 0.7045 |  | 5.37 | 1.97-14.66 | 0.0010 |
| ***Ethnicity: Other*** | 1.45 | 0.36-5.89 | 0.6063 |  | 0.64 | 0.16-2.59 | 0.5352 |  | 2.08 | 0.66-6.58 | 0.2135 |
| ***IMD*** | 0.98 | 0.95-1.02 | 0.4419 |  | 0.98 | 0.95-1.00 | 0.0700 |  | 1.01 | 0.97-1.04 | 0.7726 |
| ***North West*** | 0.76 | 0.40-1.44 | 0.4081 |  | 0.84 | 0.58-1.22 | 0.3597 |  | 0.71 | 0.38-1.34 | 0.2892 |
| ***Yorkshire & Humber*** | 0.32 | 0.04-2.30 | 0.2584 |  | 0.72 | 0.32-1.63 | 0.4346 |  | 0.35 | 0.05-2.53 | 0.3003 |
| ***East Midlands*** | 0.49 | 0.07-3.50 | 0.4744 |  | 0.63 | 0.20-1.98 | 0.4316 |  | 0.42 | 0.06-3.05 | 0.3945 |
| ***West Midlands*** | 1.49 | 0.84-2.65 | 0.1766 |  | 1.26 | 0.86-1.83 | 0.2308 |  | 1.79 | 1.05-3.05 | 0.0316 |
| ***East of England*** | 1.14 | 0.57-2.28 | 0.7065 |  | 0.96 | 0.62-1.50 | 0.8646 |  | 1.11 | 0.57-2.15 | 0.7548 |
| ***South West*** | 0.61 | 0.28-1.33 | 0.2155 |  | 0.97 | 0.66-1.43 | 0.8804 |  | 0.93 | 0.50-1.76 | 0.8352 |
| ***South Central*** | 1.08 | 0.57-2.05 | 0.8032 |  | 1.05 | 0.71-1.57 | 0.7937 |  | 0.80 | 0.38-1.65 | 0.5396 |
| ***London*** | 1.01 | 0.54-1.92 | 0.9654 |  | 1.46 | 1.03-2.06 | 0.0331 |  | 1.10 | 0.60-2.03 | 0.7537 |
| ***South East Coast*** | 1.57 | 0.90-2.75 | 0.1152 |  | 0.81 | 0.53-1.24 | 0.3351 |  | 1.18 | 0.66-2.14 | 0.5761 |
| ***BMI: Underweight*** | 1.23 | 0.68-2.23 | 0.4939 |  | 1.00 | 0.68-1.47 | 0.9914 |  | 0.97 | 0.51-1.82 | 0.9130 |
| ***BMI: Overweight*** | 0.78 | 0.47-1.29 | 0.3377 |  | 1.01 | 0.76-1.34 | 0.9491 |  | 1.10 | 0.70-1.73 | 0.6875 |
| ***BMI: Obese*** | 0.99 | 0.64-1.53 | 0.9649 |  | 1.09 | 0.83-1.42 | 0.5402 |  | 0.88 | 0.57-1.34 | 0.5508 |
| ***BMI: Missing*** | 1.82 | 0.67-4.97 | 0.2440 |  | 0.23 | 0.03-1.64 | 0.1423 |  | 1.40 | 0.51-3.83 | 0.5086 |
| ***Ex-Smoker*** | 0.93 | 0.57-1.51 | 0.7609 |  | 1.07 | 0.81-1.40 | 0.6497 |  | 1.31 | 0.84-2.04 | 0.2324 |
| ***Current Smoker*** | 0.75 | 0.42-1.36 | 0.3466 |  | 0.63 | 0.40-0.98 | 0.0416 |  | 0.71 | 0.39-1.27 | 0.2468 |
| ***Smoking: Missing*** | 2.33 | 0.32-16.77 | 0.4002 |  | 4.33 | 1.38-13.53 | 0.0118 |  | 2.04 | 0.28-14.64 | 0.4795 |
| ***Alcohol: 1-14*** | 0.89 | 0.58-1.38 | 0.6123 |  | 0.85 | 0.66-1.11 | 0.2452 |  | 0.85 | 0.55-1.30 | 0.4472 |
| ***Alcohol: 15-42*** | 0.64 | 0.28-1.46 | 0.2883 |  | 0.84 | 0.52-1.36 | 0.4865 |  | 0.70 | 0.32-1.52 | 0.3699 |
| ***Alcohol: >42*** | 1.13 | 0.36-3.58 | 0.8362 |  | 0.85 | 0.35-2.07 | 0.7224 |  | 0.96 | 0.30-3.03 | 0.9409 |
| ***Alcohol: Missing*** | 1.36 | 0.81-2.27 | 0.2440 |  | 1.32 | 0.95-1.84 | 0.0975 |  | 1.43 | 0.86-2.35 | 0.1651 |
| ***Morbidities*** | 0.81 | 0.69-0.96 | 0.0143 |  | 0.88 | 0.81-0.97 | 0.0077 |  | 0.82 | 0.69-0.96 | 0.0150 |
| ***Prescriptions*** | 1.01 | 1.00-1.03 | 0.1025 |  | 1.02 | 1.01-1.03 | 0.0008 |  | 1.01 | 1.00-1.03 | 0.0631 |
| ***Hospitalisations*** | 0.91 | 0.57-1.45 | 0.6812 |  | 0.96 | 0.75-1.22 | 0.7263 |  | 0.86 | 0.52-1.43 | 0.5628 |
| ***Duration of diabetes (years)*** | 0.99 | 0.95-1.04 | 0.7428 |  | 1.02 | 1.00-1.04 | 0.0844 |  | 1.01 | 0.97-1.05 | 0.6949 |
| ***Complications*** | 1.82 | 1.56-2.13 | <0.0001 |  | 1.65 | 1.50-1.82 | <0.0001 |  | 1.74 | 1.50-2.02 | <0.0001 |
| ***Glucose lowering therapies*** | 1.56 | 1.29-1.88 | <0.0001 |  | 1.56 | 1.39-1.75 | <0.0001 |  | 1.40 | 1.19-1.65 | <0.0001 |
| ***Insulin prescription*** | 3.46 | 2.05-5.84 | <0.0001 |  | 2.92 | 2.16-3.93 | <0.0001 |  | 1.94 | 1.09-3.44 | 0.0238 |

Study sizes across exposures after 1:1 propensity score matching are found in Supplementary Table 5, as they are the same between univariate and multivariate analyses.

Additional File 1: Table S12: Univariate hazard ratios (with corresponding 95% CIs and p-values) for risk of sight-threatening diabetic retinopathy by each covariate across NDA and QOF exposure definitions after 1:1 propensity score matching.

|  | **Exposure Definition** | | | | | | | | | | |
| --- | --- | --- | --- | --- | --- | --- | --- | --- | --- | --- | --- |
|  | **Achieve All QOF Targets** | | |  | **Meet All NDA Processes** | | |  | **Achieve All QOF & NDA Targets** | | |
|  | **Hazard Ratio** | **95% CI** | **p** |  | **Hazard Ratio** | **95% CI** | **p** |  | **Hazard Ratio** | **95% CI** | **p** |
| ***Exposure*** | 0.75 | 0.62-0.90 | 0.0023 |  | 0.85 | 0.74-0.99 | 0.0372 |  | 0.75 | 0.58-0.97 | 0.0296 |
| ***Age*** | 1.00 | 0.99-1.00 | 0.3823 |  | 0.98 | 0.98-0.99 | <0.0001 |  | 0.99 | 0.98-1.00 | 0.1294 |
| ***Sex: Female*** | 0.92 | 0.76-1.11 | 0.3837 |  | 0.87 | 0.75-1.02 | 0.0823 |  | 0.96 | 0.74-1.25 | 0.7524 |
| ***Ethnicity: Asian*** | 1.02 | 0.69-1.51 | 0.9167 |  | 1.43 | 1.10-1.87 | 0.0085 |  | 0.96 | 0.52-1.76 | 0.8933 |
| ***Ethnicity: Black*** | 1.57 | 0.88-2.79 | 0.1232 |  | 1.54 | 0.98-2.43 | 0.0621 |  | 2.68 | 1.32-5.42 | 0.0062 |
| ***Ethnicity: Mixed*** | 2.33 | 1.04-5.22 | 0.0394 |  | 2.13 | 1.17-3.86 | 0.0129 |  | 1.41 | 0.35-5.69 | 0.6258 |
| ***Ethnicity: Other*** | 1.01 | 0.42-2.45 | 0.9753 |  | 0.66 | 0.27-1.58 | 0.3466 |  | 1.68 | 0.62-4.51 | 0.3051 |
| ***IMD*** | 1.00 | 0.98-1.01 | 0.7757 |  | 1.00 | 0.99-1.01 | 0.9604 |  | 0.99 | 0.97-1.02 | 0.6116 |
| ***North West*** | 1.08 | 0.86-1.37 | 0.5071 |  | 1.16 | 0.96-1.40 | 0.1145 |  | 1.09 | 0.78-1.51 | 0.6078 |
| ***Yorkshire & Humber*** | 0.84 | 0.50-1.41 | 0.5147 |  | 0.73 | 0.46-1.15 | 0.1750 |  | 0.59 | 0.24-1.43 | 0.2435 |
| ***East Midlands*** | 0.90 | 0.47-1.74 | 0.7544 |  | 1.07 | 0.66-1.73 | 0.7830 |  | 1.00 | 0.41-2.43 | 0.9950 |
| ***West Midlands*** | 0.99 | 0.74-1.31 | 0.9355 |  | 1.08 | 0.87-1.35 | 0.4831 |  | 1.15 | 0.79-1.67 | 0.4657 |
| ***East of England*** | 0.79 | 0.57-1.11 | 0.1776 |  | 1.01 | 0.80-1.29 | 0.9066 |  | 0.77 | 0.48-1.23 | 0.2663 |
| ***South West*** | 1.16 | 0.90-1.51 | 0.2543 |  | 1.00 | 0.81-1.24 | 0.9991 |  | 0.97 | 0.66-1.43 | 0.8839 |
| ***South Central*** | 1.04 | 0.78-1.37 | 0.7957 |  | 0.82 | 0.64-1.05 | 0.1155 |  | 1.21 | 0.83-1.75 | 0.3246 |
| ***London*** | 1.05 | 0.80-1.38 | 0.7054 |  | 1.00 | 0.80-1.24 | 0.9813 |  | 1.07 | 0.73-1.56 | 0.7316 |
| ***South East Coast*** | 1.03 | 0.78-1.36 | 0.8361 |  | 1.07 | 0.86-1.33 | 0.5326 |  | 0.99 | 0.66-1.48 | 0.9696 |
| ***BMI: Underweight*** | 1.36 | 1.08-1.71 | 0.0094 |  | 1.14 | 0.92-1.40 | 0.2312 |  | 1.18 | 0.84-1.67 | 0.3329 |
| ***BMI: Overweight*** | 0.90 | 0.74-1.09 | 0.2746 |  | 0.96 | 0.82-1.12 | 0.6175 |  | 0.87 | 0.66-1.14 | 0.3168 |
| ***BMI: Obese*** | 0.93 | 0.77-1.12 | 0.4386 |  | 1.00 | 0.86-1.16 | 0.9860 |  | 1.06 | 0.82-1.38 | 0.6562 |
| ***BMI: Missing*** | 0.56 | 0.14-2.27 | 0.4203 |  | 0.49 | 0.16-1.53 | 0.2221 |  | 0.67 | 0.09-4.74 | 0.6843 |
| ***Ex-Smoker*** | 1.05 | 0.87-1.27 | 0.5997 |  | 0.94 | 0.80-1.09 | 0.3927 |  | 1.09 | 0.84-1.42 | 0.5045 |
| ***Current Smoker*** | 0.90 | 0.68-1.20 | 0.4887 |  | 0.87 | 0.69-1.09 | 0.2176 |  | 0.91 | 0.62-1.36 | 0.6554 |
| ***Smoking: Missing*** | N/A | N/A | N/A |  | N/A | N/A | N/A |  | N/A | N/A | N/A |
| ***Alcohol: 1-14*** | 0.93 | 0.77-1.13 | 0.4657 |  | 0.93 | 0.80-1.08 | 0.3378 |  | 0.96 | 0.73-1.25 | 0.7628 |
| ***Alcohol: 15-42*** | 0.79 | 0.57-1.12 | 0.1868 |  | 0.76 | 0.58-1.00 | 0.0477 |  | 0.66 | 0.40-1.10 | 0.1087 |
| ***Alcohol: >42*** | 0.79 | 0.38-1.67 | 0.5413 |  | 0.77 | 0.43-1.41 | 0.4008 |  | 0.21 | 0.03-1.52 | 0.1236 |
| ***Alcohol: Missing*** | 0.95 | 0.71-1.28 | 0.7381 |  | 1.20 | 0.96-1.49 | 0.1180 |  | 1.08 | 0.70-1.64 | 0.7371 |
| ***Morbidities*** | 1.02 | 0.96-1.08 | 0.4933 |  | 0.98 | 0.93-1.03 | 0.3555 |  | 0.99 | 0.91-1.07 | 0.7855 |
| ***Prescriptions*** | 1.02 | 1.01-1.03 | <0.0001 |  | 1.02 | 1.02-1.03 | <0.0001 |  | 1.02 | 1.01-1.03 | 0.0005 |
| ***Hospitalisations*** | 1.00 | 0.81-1.24 | 0.9885 |  | 0.96 | 0.79-1.17 | 0.6925 |  | 0.85 | 0.57-1.27 | 0.4318 |
| ***Duration of diabetes (years)*** | 1.01 | 0.99-1.03 | 0.2941 |  | 1.01 | 1.00-1.03 | 0.0314 |  | 1.01 | 0.98-1.03 | 0.5446 |
| ***Complications*** | 1.75 | 1.64-1.87 | <0.0001 |  | 1.72 | 1.63-1.81 | <0.0001 |  | 1.72 | 1.57-1.89 | <0.0001 |
| ***Glucose lowering therapies*** | 1.55 | 1.41-1.69 | <0.0001 |  | 1.55 | 1.45-1.66 | <0.0001 |  | 1.69 | 1.50-1.92 | <0.0001 |
| ***Insulin prescription*** | 2.83 | 2.18-3.67 | <0.0001 |  | 2.54 | 2.12-3.04 | <0.0001 |  | 2.99 | 2.06-4.34 | <0.0001 |

Study sizes across exposures after 1:1 propensity score matching are found in Supplementary Table 6, as they are the same between univariate and multivariate analyses. N/A indicates that the sample after propensity score matching did not contain observations for the covariate.

Additional File 1: Table S13: Multivariate hazard ratios (with corresponding 95% CIs and p-values) for risk of sight-threatening diabetic retinopathy by each covariate across QOF exposure definitions after 1:1 propensity score matching, including the adjusted study size (n) and C-statistic (also with corresponding 95% CI).

|  | **Exposure Definition** | | | | | | | | | | |
| --- | --- | --- | --- | --- | --- | --- | --- | --- | --- | --- | --- |
|  | **Achieve HbA1c QOF**  **Target** | | |  | **Achieve Blood Pressure QOF Target** | | |  | **Achieve Cholesterol QOF**  **Target** | | |
|  | **Hazard Ratio** | **95% CI** | **p** |  | **Hazard Ratio** | **95% CI** | **p** |  | **Hazard Ratio** | **95% CI** | **p** |
| ***Exposure*** | 0.74 | 0.62-0.87 | 0.0002 |  | 0.78 | 0.67-0.91 | 0.0015 |  | 0.82 | 0.67-0.99 | 0.0428 |
| ***Age*** | 0.98 | 0.97-0.98 | <0.0001 |  | 0.97 | 0.97-0.98 | <0.0001 |  | 0.97 | 0.96-0.98 | <0.0001 |
| ***Sex: Female*** | 1.50 | 1.25-1.79 | <0.0001 |  | 1.48 | 1.25-1.75 | <0.0001 |  | 1.56 | 1.26-1.94 | 0.0001 |
| ***Ethnicity: Asian*** | 1.08 | 0.81-1.45 | 0.5841 |  | 0.99 | 0.74-1.31 | 0.9343 |  | 0.84 | 0.56-1.24 | 0.3714 |
| ***Ethnicity: Black*** | 1.27 | 0.84-1.92 | 0.2484 |  | 1.29 | 0.88-1.89 | 0.1902 |  | 1.71 | 1.12-2.60 | 0.0121 |
| ***Ethnicity: Mixed*** | 1.17 | 0.52-2.62 | 0.7049 |  | 1.37 | 0.73-2.56 | 0.3312 |  | 1.65 | 0.73-3.71 | 0.2292 |
| ***Ethnicity: Other*** | 0.96 | 0.47-1.94 | 0.9095 |  | 1.01 | 0.54-1.90 | 0.9778 |  | 0.90 | 0.39-2.03 | 0.7906 |
| ***IMD*** | 1.00 | 0.99-1.02 | 0.9453 |  | 0.99 | 0.98-1.01 | 0.2409 |  | 0.99 | 0.97-1.01 | 0.2368 |
| ***North West*** | 1.75 | 0.89-3.46 | 0.1051 |  | 1.88 | 0.99-3.59 | 0.0542 |  | 1.69 | 0.78-3.66 | 0.1843 |
| ***Yorkshire & Humber*** | 1.42 | 0.65-3.10 | 0.3820 |  | 1.52 | 0.72-3.18 | 0.2687 |  | 1.21 | 0.47-3.08 | 0.6901 |
| ***East Midlands*** | 1.31 | 0.55-3.10 | 0.5467 |  | 1.49 | 0.67-3.33 | 0.3289 |  | 1.12 | 0.40-3.09 | 0.8312 |
| ***West Midlands*** | 1.40 | 0.70-2.81 | 0.3466 |  | 1.62 | 0.84-3.13 | 0.1534 |  | 1.45 | 0.66-3.21 | 0.3572 |
| ***East of England*** | 1.78 | 0.89-3.59 | 0.1046 |  | 1.77 | 0.91-3.43 | 0.0927 |  | 1.31 | 0.58-2.96 | 0.5088 |
| ***South West*** | 1.56 | 0.78-3.11 | 0.2098 |  | 1.94 | 1.01-3.72 | 0.0452 |  | 1.43 | 0.65-3.15 | 0.3758 |
| ***South Central*** | 1.33 | 0.66-2.69 | 0.4249 |  | 1.31 | 0.67-2.55 | 0.4334 |  | 1.27 | 0.57-2.85 | 0.5551 |
| ***London*** | 1.58 | 0.79-3.15 | 0.1968 |  | 1.76 | 0.91-3.38 | 0.0913 |  | 1.43 | 0.65-3.15 | 0.3728 |
| ***South East Coast*** | 1.78 | 0.89-3.56 | 0.1027 |  | 1.80 | 0.94-3.48 | 0.0781 |  | 1.56 | 0.71-3.44 | 0.2704 |
| ***BMI: Underweight*** | 1.38 | 0.51-3.79 | 0.5261 |  | 1.63 | 0.60-4.43 | 0.3400 |  | 1.25 | 0.39-4.01 | 0.7087 |
| ***BMI: Overweight*** | 1.06 | 0.39-2.85 | 0.9156 |  | 1.25 | 0.46-3.36 | 0.6631 |  | 1.13 | 0.36-3.57 | 0.8342 |
| ***BMI: Obese*** | 1.03 | 0.38-2.77 | 0.9566 |  | 1.08 | 0.40-2.90 | 0.8821 |  | 0.96 | 0.31-3.03 | 0.9459 |
| ***BMI: Missing*** | 0.98 | 0.28-3.49 | 0.9767 |  | 0.90 | 0.25-3.19 | 0.8651 |  | 1.02 | 0.24-4.29 | 0.9800 |
| ***Ex-Smoker*** | 0.86 | 0.71-1.03 | 0.1088 |  | 0.88 | 0.74-1.05 | 0.1537 |  | 0.86 | 0.68-1.08 | 0.1953 |
| ***Current Smoker*** | 0.69 | 0.54-0.88 | 0.0028 |  | 0.70 | 0.56-0.88 | 0.0022 |  | 0.68 | 0.51-0.91 | 0.0084 |
| ***Smoking: Missing*** | 0.64 | 0.09-4.57 | 0.6562 |  | 1.24 | 0.31-5.00 | 0.7591 |  | 2.09 | 0.52-8.48 | 0.3000 |
| ***Alcohol: 1-14*** | 1.00 | 0.80-1.26 | 0.9756 |  | 0.98 | 0.80-1.21 | 0.8645 |  | 0.88 | 0.68-1.15 | 0.3549 |
| ***Alcohol: 15-42*** | 0.86 | 0.60-1.23 | 0.3971 |  | 0.77 | 0.56-1.08 | 0.1289 |  | 0.69 | 0.44-1.08 | 0.1086 |
| ***Alcohol: >42*** | 0.93 | 0.51-1.71 | 0.8169 |  | 0.94 | 0.55-1.59 | 0.8127 |  | 0.87 | 0.44-1.69 | 0.6772 |
| ***Alcohol: Missing*** | 1.16 | 0.88-1.52 | 0.3007 |  | 1.13 | 0.87-1.47 | 0.3485 |  | 1.11 | 0.81-1.54 | 0.5176 |
| ***Morbidities*** | 0.78 | 0.73-0.83 | <0.0001 |  | 0.80 | 0.76-0.85 | <0.0001 |  | 0.82 | 0.76-0.89 | <0.0001 |
| ***Prescriptions*** | 1.00 | 0.99-1.01 | 0.7353 |  | 1.00 | 0.99-1.01 | 0.7567 |  | 1.00 | 0.99-1.01 | 0.9258 |
| ***Hospitalisations*** | 0.94 | 0.79-1.13 | 0.5242 |  | 0.87 | 0.73-1.04 | 0.1338 |  | 0.92 | 0.76-1.12 | 0.4087 |
| ***Duration of diabetes (years)*** | 0.99 | 0.98-1.01 | 0.3420 |  | 1.00 | 0.99-1.02 | 0.7880 |  | 1.00 | 0.98-1.02 | 0.9861 |
| ***Complications*** | 2.03 | 1.90-2.18 | <0.0001 |  | 2.10 | 1.98-2.24 | <0.0001 |  | 2.13 | 1.96-2.32 | <0.0001 |
| ***Glucose lowering therapies*** | 1.29 | 1.17-1.42 | <0.0001 |  | 1.33 | 1.22-1.46 | <0.0001 |  | 1.35 | 1.20-1.51 | <0.0001 |
| ***Insulin prescription*** | 1.38 | 1.13-1.70 | 0.0020 |  | 1.26 | 1.03-1.55 | 0.0266 |  | 1.34 | 1.03-1.75 | 0.0307 |
|  |  |  |  |  |  |  |  |  |  |  |  |
|  | **Value** | **95% CI (lower)** | **95% CI (upper)** |  | **Value** | **95% CI (lower)** | **95% CI (upper)** |  | **Value** | **95% CI (lower)** | **95% CI (upper)** |
| ***n after matching*** | 37,182 |  |  |  | 50,426 |  |  |  | 30,978 |  |  |
| ***C-statistic*** | 0.7647 | 0.7645 | 0.7648 |  | 0.7837 | 0.7835 | 0.7838 |  | 0.7941 | 0.7938 | 0.7943 |

Reference groups for categorical covariates include: white (ethnicity); North East (region); normal weight (BMI); non-smoker (smoking status); 0 units (alcohol consumption); and no insulin prescription (insulin use).

Additional File 1: Table S14: Multivariate hazard ratios (with corresponding 95% CIs and p-values) for risk of sight-threatening diabetic retinopathy by each covariate across NDA exposure definitions after 1:1 propensity score matching, including the adjusted study size (n) and C-statistic (also with corresponding 95% CI).

|  | **Exposure Definition** | | | | | | | | | | |
| --- | --- | --- | --- | --- | --- | --- | --- | --- | --- | --- | --- |
|  | **Meet 4-6 NDA Processes (vs. Meet 0-3 NDA Processes)** | | |  | **Meet 7-9 NDA Processes (vs. Meet 0-3 NDA Processes)** | | |  | **Meet 7-9 NDA Processes (vs. Meet 4-6 NDA Processes)** | | |
|  | **Hazard Ratio** | **95% CI** | **p** |  | **Hazard Ratio** | **95% CI** | **p** |  | **Hazard Ratio** | **95% CI** | **p** |
| ***Exposure*** | 1.14 | 0.73-1.78 | 0.5715 |  | 0.72 | 0.55-0.94 | 0.0166 |  | 1.14 | 0.73-1.78 | 0.5720 |
| ***Age*** | 0.98 | 0.96-1.00 | 0.0140 |  | 0.97 | 0.96-0.98 | <0.0001 |  | 0.97 | 0.95-0.99 | 0.0010 |
| ***Sex: Female*** | 1.47 | 0.89-2.44 | 0.1362 |  | 1.63 | 1.21-2.19 | 0.0013 |  | 1.37 | 0.83-2.24 | 0.2166 |
| ***Ethnicity: Asian*** | 0.78 | 0.33-1.83 | 0.5659 |  | 0.92 | 0.55-1.52 | 0.7396 |  | 0.52 | 0.18-1.50 | 0.2250 |
| ***Ethnicity: Black*** | 1.67 | 0.72-3.87 | 0.2326 |  | 2.23 | 1.35-3.67 | 0.0016 |  | 1.65 | 0.75-3.66 | 0.2154 |
| ***Ethnicity: Mixed*** | 2.16 | 0.51-9.23 | 0.2991 |  | 0.58 | 0.08-4.19 | 0.5918 |  | 3.45 | 1.20-9.95 | 0.0219 |
| ***Ethnicity: Other*** | 1.54 | 0.35-6.70 | 0.5669 |  | 0.63 | 0.15-2.61 | 0.5262 |  | 2.01 | 0.60-6.74 | 0.2567 |
| ***IMD*** | 0.99 | 0.95-1.03 | 0.5280 |  | 0.97 | 0.95-1.00 | 0.0230 |  | 1.01 | 0.97-1.05 | 0.5424 |
| ***North West*** | 1.47 | 0.19-11.56 | 0.7122 |  | 1.23 | 0.38-4.02 | 0.7312 |  | 1.84 | 0.23-14.55 | 0.5631 |
| ***Yorkshire & Humber*** | 0.58 | 0.04-9.53 | 0.7065 |  | 0.94 | 0.23-3.76 | 0.9247 |  | 0.81 | 0.05-13.36 | 0.8851 |
| ***East Midlands*** | 0.55 | 0.03-8.92 | 0.6712 |  | 0.53 | 0.10-2.65 | 0.4367 |  | 0.54 | 0.03-8.99 | 0.6688 |
| ***West Midlands*** | 1.98 | 0.25-15.53 | 0.5141 |  | 1.64 | 0.50-5.37 | 0.4178 |  | 3.45 | 0.44-26.77 | 0.2360 |
| ***East of England*** | 1.91 | 0.23-15.50 | 0.5455 |  | 1.31 | 0.39-4.43 | 0.6606 |  | 2.49 | 0.31-20.13 | 0.3934 |
| ***South West*** | 1.17 | 0.14-9.70 | 0.8863 |  | 1.31 | 0.40-4.31 | 0.6622 |  | 2.05 | 0.26-16.29 | 0.4980 |
| ***South Central*** | 1.63 | 0.20-13.05 | 0.6468 |  | 1.30 | 0.39-4.34 | 0.6654 |  | 1.87 | 0.23-15.53 | 0.5616 |
| ***London*** | 1.77 | 0.22-14.01 | 0.5864 |  | 1.89 | 0.58-6.15 | 0.2882 |  | 2.38 | 0.30-18.66 | 0.4102 |
| ***South East Coast*** | 2.53 | 0.32-19.72 | 0.3766 |  | 1.13 | 0.34-3.77 | 0.8469 |  | 2.70 | 0.34-21.35 | 0.3477 |
| ***BMI: Underweight*** | 2.48 | 0.30-20.11 | 0.3966 |  | N/A | N/A | N/A |  | 1.46 | 0.18-11.70 | 0.7189 |
| ***BMI: Overweight*** | 1.21 | 0.15-9.54 | 0.8531 |  | N/A | N/A | N/A |  | 1.34 | 0.18-10.19 | 0.7746 |
| ***BMI: Obese*** | 1.39 | 0.18-10.95 | 0.7525 |  | N/A | N/A | N/A |  | 1.09 | 0.14-8.24 | 0.9312 |
| ***BMI: Missing*** | 4.33 | 0.44-42.63 | 0.2094 |  | N/A | N/A | N/A |  | 2.75 | 0.29-25.88 | 0.3755 |
| ***Ex-Smoker*** | 0.91 | 0.52-1.56 | 0.7219 |  | 0.98 | 0.72-1.33 | 0.8740 |  | 1.34 | 0.81-2.23 | 0.2567 |
| ***Current Smoker*** | 0.62 | 0.33-1.19 | 0.1519 |  | 0.52 | 0.32-0.83 | 0.0068 |  | 0.55 | 0.29-1.07 | 0.0774 |
| ***Smoking: Missing*** | 3.09 | 0.41-23.18 | 0.2719 |  | 5.37 | 1.68-17.10 | 0.0045 |  | 1.09 | 0.14-8.49 | 0.9334 |
| ***Alcohol: 1-14*** | 1.00 | 0.53-1.90 | 0.9980 |  | 0.94 | 0.65-1.36 | 0.7442 |  | 0.94 | 0.50-1.78 | 0.8560 |
| ***Alcohol: 15-42*** | 0.81 | 0.30-2.21 | 0.6820 |  | 0.92 | 0.51-1.65 | 0.7818 |  | 0.73 | 0.28-1.89 | 0.5175 |
| ***Alcohol: >42*** | 1.19 | 0.33-4.34 | 0.7897 |  | 0.80 | 0.31-2.08 | 0.6522 |  | 1.20 | 0.33-4.34 | 0.7856 |
| ***Alcohol: Missing*** | 1.05 | 0.51-2.13 | 0.9031 |  | 1.10 | 0.71-1.71 | 0.6595 |  | 1.33 | 0.65-2.72 | 0.4388 |
| ***Morbidities*** | 0.63 | 0.52-0.77 | <0.0001 |  | 0.73 | 0.65-0.81 | <0.0001 |  | 0.66 | 0.54-0.80 | <0.0001 |
| ***Prescriptions*** | 1.00 | 0.97-1.03 | 0.8874 |  | 1.00 | 0.98-1.02 | 0.8357 |  | 1.01 | 0.98-1.03 | 0.6081 |
| ***Hospitalisations*** | 0.79 | 0.47-1.33 | 0.3803 |  | 0.87 | 0.68-1.13 | 0.3046 |  | 0.84 | 0.49-1.45 | 0.5424 |
| ***Duration of diabetes (years)*** | 0.98 | 0.94-1.03 | 0.4313 |  | 1.02 | 0.99-1.04 | 0.1856 |  | 1.01 | 0.96-1.05 | 0.7964 |
| ***Complications*** | 2.47 | 2.05-2.99 | <0.0001 |  | 2.24 | 1.99-2.54 | <0.0001 |  | 2.23 | 1.87-2.66 | <0.0001 |
| ***Glucose lowering therapies*** | 1.29 | 1.00-1.68 | 0.0534 |  | 1.27 | 1.09-1.48 | 0.0022 |  | 1.32 | 1.05-1.65 | 0.0178 |
| ***Insulin prescription*** | 2.06 | 1.11-3.81 | 0.0216 |  | 1.57 | 1.09-2.25 | 0.0142 |  | 1.05 | 0.54-2.07 | 0.8798 |
|  |  |  |  |  |  |  |  |  |  |  |  |
|  | **Value** | **95% CI (lower)** | **95% CI (upper)** |  | **Value** | **95% CI (lower)** | **95% CI (upper)** |  | **Value** | **95% CI (lower)** | **95% CI (upper)** |
| ***n after matching*** | 5,438 |  |  |  | 14,052 |  |  |  | 5,484 |  |  |
| ***C-statistic*** | 0.8467 | 0.8461 | 0.8472 |  | 0.7966 | 0.7961 | 0.7970 |  | 0.8434 | 0.8428 | 0.8440 |

Reference groups for categorical covariates include: white (ethnicity); North East (region); normal weight (BMI); non-smoker (smoking status); 0 units (alcohol consumption); and no insulin prescription (insulin use).

Additional File 1: Table S15: Multivariate hazard ratios (with corresponding 95% CIs and p-values) for risk of sight-threatening diabetic retinopathy by each covariate across QOF and NDA exposure definitions after 1:1 propensity score matching, including the adjusted study size (n), and C-statistic (also with corresponding 95% CI).

|  | **Exposure Definition** | | | | | | | | | | |
| --- | --- | --- | --- | --- | --- | --- | --- | --- | --- | --- | --- |
|  | **Achieve All QOF Targets** | | |  | **Meet All NDA Processes** | | |  | **Achieve All QOF & NDA Targets** | | |
|  | **Hazard Ratio** | **95% CI** | **p** |  | **Hazard Ratio** | **95% CI** | **p** |  | **Hazard Ratio** | **95% CI** | **p** |
| ***Exposure*** | 0.77 | 0.64-0.93 | 0.0065 |  | 0.88 | 0.76-1.03 | 0.1083 |  | 0.81 | 0.62-1.06 | 0.1187 |
| ***Age*** | 0.98 | 0.97-0.99 | 0.0003 |  | 0.97 | 0.96-0.98 | <0.0001 |  | 0.98 | 0.97-0.99 | 0.0054 |
| ***Sex: Female*** | 1.55 | 1.25-1.91 | 0.0001 |  | 1.51 | 1.28-1.79 | <0.0001 |  | 1.71 | 1.27-2.30 | 0.0004 |
| ***Ethnicity: Asian*** | 0.82 | 0.54-1.25 | 0.3647 |  | 1.01 | 0.75-1.35 | 0.9699 |  | 0.75 | 0.39-1.43 | 0.3784 |
| ***Ethnicity: Black*** | 1.32 | 0.74-2.36 | 0.3485 |  | 1.25 | 0.78-1.98 | 0.3502 |  | 2.14 | 1.04-4.41 | 0.0386 |
| ***Ethnicity: Mixed*** | 1.96 | 0.87-4.42 | 0.1058 |  | 1.68 | 0.92-3.07 | 0.0888 |  | 1.36 | 0.33-5.51 | 0.6695 |
| ***Ethnicity: Other*** | 1.07 | 0.44-2.62 | 0.8749 |  | 0.62 | 0.26-1.50 | 0.2866 |  | 1.75 | 0.64-4.80 | 0.2737 |
| ***IMD*** | 1.00 | 0.98-1.01 | 0.7690 |  | 1.00 | 0.98-1.01 | 0.8468 |  | 1.00 | 0.97-1.02 | 0.7070 |
| ***North West*** | 2.50 | 1.01-6.17 | 0.0465 |  | 2.29 | 1.16-4.49 | 0.0164 |  | 3.03 | 0.73-12.52 | 0.1259 |
| ***Yorkshire & Humber*** | 1.98 | 0.72-5.46 | 0.1873 |  | 1.45 | 0.65-3.21 | 0.3619 |  | 1.73 | 0.33-8.94 | 0.5139 |
| ***East Midlands*** | 2.04 | 0.68-6.09 | 0.2029 |  | 1.85 | 0.82-4.15 | 0.1373 |  | 2.81 | 0.54-14.54 | 0.2175 |
| ***West Midlands*** | 2.20 | 0.88-5.51 | 0.0930 |  | 2.01 | 1.01-4.00 | 0.0464 |  | 3.18 | 0.76-13.32 | 0.1142 |
| ***East of England*** | 1.92 | 0.75-4.90 | 0.1752 |  | 1.96 | 0.97-3.93 | 0.0593 |  | 2.28 | 0.53-9.89 | 0.2695 |
| ***South West*** | 2.55 | 1.02-6.34 | 0.0442 |  | 1.89 | 0.95-3.76 | 0.0681 |  | 2.71 | 0.65-11.40 | 0.1732 |
| ***South Central*** | 2.33 | 0.93-5.87 | 0.0716 |  | 1.57 | 0.78-3.16 | 0.2065 |  | 3.16 | 0.75-13.33 | 0.1170 |
| ***London*** | 2.47 | 0.99-6.15 | 0.0529 |  | 1.92 | 0.97-3.82 | 0.0618 |  | 2.87 | 0.68-12.01 | 0.1496 |
| ***South East Coast*** | 2.40 | 0.96-6.04 | 0.0621 |  | 2.05 | 1.03-4.07 | 0.0416 |  | 2.90 | 0.68-12.28 | 0.1492 |
| ***BMI: Underweight*** | 1.29 | 0.47-3.52 | 0.6200 |  | 1.84 | 0.68-5.01 | 0.2319 |  | 2.53 | 0.35-18.43 | 0.3607 |
| ***BMI: Overweight*** | 0.85 | 0.31-2.29 | 0.7430 |  | 1.40 | 0.52-3.77 | 0.5058 |  | 1.74 | 0.24-12.53 | 0.5839 |
| ***BMI: Obese*** | 0.77 | 0.29-2.10 | 0.6158 |  | 1.21 | 0.45-3.24 | 0.7115 |  | 1.72 | 0.24-12.39 | 0.5913 |
| ***BMI: Missing*** | 0.58 | 0.11-3.15 | 0.5239 |  | 0.74 | 0.16-3.29 | 0.6875 |  | 1.57 | 0.10-25.24 | 0.7512 |
| ***Ex-Smoker*** | 0.94 | 0.76-1.16 | 0.5833 |  | 0.86 | 0.73-1.02 | 0.0869 |  | 1.05 | 0.78-1.41 | 0.7551 |
| ***Current Smoker*** | 0.71 | 0.52-0.98 | 0.0348 |  | 0.64 | 0.51-0.82 | 0.0003 |  | 0.82 | 0.54-1.27 | 0.3801 |
| ***Smoking: Missing*** | N/A | N/A | N/A |  | N/A | N/A | N/A |  | N/A | N/A | N/A |
| ***Alcohol: 1-14*** | 0.85 | 0.66-1.08 | 0.1806 |  | 0.93 | 0.76-1.14 | 0.4866 |  | 0.82 | 0.59-1.16 | 0.2592 |
| ***Alcohol: 15-42*** | 0.74 | 0.50-1.11 | 0.1465 |  | 0.77 | 0.55-1.06 | 0.1127 |  | 0.60 | 0.34-1.09 | 0.0945 |
| ***Alcohol: >42*** | 0.72 | 0.33-1.57 | 0.4119 |  | 0.70 | 0.38-1.31 | 0.2623 |  | 0.20 | 0.03-1.48 | 0.1154 |
| ***Alcohol: Missing*** | 0.83 | 0.58-1.17 | 0.2843 |  | 1.01 | 0.76-1.33 | 0.9650 |  | 0.84 | 0.51-1.39 | 0.4950 |
| ***Morbidities*** | 0.81 | 0.75-0.87 | <0.0001 |  | 0.80 | 0.76-0.85 | <0.0001 |  | 0.79 | 0.72-0.88 | <0.0001 |
| ***Prescriptions*** | 1.00 | 0.99-1.02 | 0.7857 |  | 1.01 | 1.00-1.02 | 0.1269 |  | 0.99 | 0.96-1.01 | 0.2588 |
| ***Hospitalisations*** | 0.86 | 0.68-1.08 | 0.1846 |  | 0.81 | 0.66-1.00 | 0.0555 |  | 0.72 | 0.48-1.09 | 0.1183 |
| ***Duration of diabetes (years)*** | 1.00 | 0.98-1.02 | 0.8935 |  | 1.01 | 0.99-1.02 | 0.4335 |  | 1.00 | 0.97-1.02 | 0.7603 |
| ***Complications*** | 2.08 | 1.92-2.25 | <0.0001 |  | 2.12 | 1.99-2.27 | <0.0001 |  | 2.10 | 1.87-2.35 | <0.0001 |
| ***Glucose lowering therapies*** | 1.34 | 1.19-1.51 | <0.0001 |  | 1.29 | 1.18-1.40 | <0.0001 |  | 1.54 | 1.30-1.83 | <0.0001 |
| ***Insulin prescription*** | 1.46 | 1.09-1.95 | 0.0109 |  | 1.23 | 1.00-1.51 | 0.0547 |  | 1.38 | 0.90-2.10 | 0.1354 |
|  |  |  |  |  |  |  |  |  |  |  |  |
|  | **Value** | **95% CI (lower)** | **95% CI (upper)** |  | **Value** | **95% CI (lower)** | **95% CI (upper)** |  | **Value** | **95% CI (lower)** | **95% CI (upper)** |
| ***n after matching*** | 40,220 |  |  |  | 49,602 |  |  |  | 19,944 |  |  |
| ***C-statistic*** | 0.7823 | 0.7820 | 0.7825 |  | 0.7775 | 0.7774 | 0.7777 |  | 0.7966 | 0.7963 | 0.7970 |

Reference groups for categorical covariates include: white (ethnicity); North East (region); normal weight (BMI); non-smoker (smoking status); 0 units (alcohol consumption); and no insulin prescription (insulin use). N/A indicates that the sample after propensity score matching did not contain observations for the covariate.

Additional File 1: Table S16: Univariate hazard ratios (with corresponding 95% CIs and p-values) for risk of sight-threatening diabetic retinopathy by each covariate across QOF exposure definitions, *among those who meet all other QOF targets*, after 1:1 propensity score matching.

|  | **Exposure Definition** | | | | | | | | | | |
| --- | --- | --- | --- | --- | --- | --- | --- | --- | --- | --- | --- |
|  | **Achieve HBA1c QOF**  **Target** | | |  | **Achieve Blood Pressure QOF Target** | | |  | **Achieve Cholesterol QOF**  **Target** | | |
|  | **Hazard Ratio** | **95% CI** | **p** |  | **Hazard Ratio** | **95% CI** | **p** |  | **Hazard Ratio** | **95% CI** | **p** |
| ***Exposure*** | 0.59 | 0.45-0.77 | 0.0001 |  | 0.93 | 0.73-1.18 | 0.5316 |  | 1.31 | 0.85-2.01 | 0.2211 |
| ***Age*** | 0.99 | 0.98-1.00 | 0.0516 |  | 1.00 | 0.99-1.01 | 0.4872 |  | 1.00 | 0.98-1.02 | 0.8603 |
| ***Sex: Female*** | 1.00 | 0.77-1.31 | 0.9945 |  | 0.83 | 0.65-1.06 | 0.1349 |  | 0.70 | 0.46-1.07 | 0.0977 |
| ***Ethnicity: Asian*** | 0.96 | 0.60-1.53 | 0.8546 |  | 1.01 | 0.62-1.65 | 0.9732 |  | 0.90 | 0.33-2.45 | 0.8323 |
| ***Ethnicity: Black*** | 1.76 | 0.87-3.56 | 0.1171 |  | 1.00 | 0.45-2.25 | 0.9938 |  | 1.83 | 0.58-5.81 | 0.3019 |
| ***Ethnicity: Mixed*** | 0.67 | 0.09-4.77 | 0.6878 |  | 1.98 | 0.74-5.32 | 0.1747 |  | N/A | N/A | N/A |
| ***Ethnicity: Other*** | N/A | N/A | N/A |  | 0.62 | 0.16-2.51 | 0.5060 |  | 0.89 | 0.12-6.36 | 0.9039 |
| ***IMD*** | 1.01 | 0.98-1.03 | 0.6553 |  | 0.98 | 0.96-1.01 | 0.1694 |  | 0.98 | 0.94-1.01 | 0.2257 |
| ***North West*** | 1.21 | 0.88-1.67 | 0.2419 |  | 1.14 | 0.84-1.54 | 0.3930 |  | 1.72 | 1.07-2.78 | 0.0260 |
| ***Yorkshire & Humber*** | 0.93 | 0.46-1.89 | 0.8493 |  | 0.38 | 0.14-1.03 | 0.0573 |  | 0.62 | 0.15-2.53 | 0.5068 |
| ***East Midlands*** | 1.19 | 0.53-2.67 | 0.6813 |  | 1.54 | 0.79-3.00 | 0.2002 |  | 1.62 | 0.51-5.12 | 0.4136 |
| ***West Midlands*** | 1.02 | 0.68-1.52 | 0.9245 |  | 0.87 | 0.59-1.29 | 0.4897 |  | 0.95 | 0.49-1.83 | 0.8750 |
| ***East of England*** | 1.16 | 0.77-1.74 | 0.4854 |  | 0.80 | 0.52-1.23 | 0.3093 |  | 0.67 | 0.29-1.54 | 0.3457 |
| ***South West*** | 0.98 | 0.67-1.44 | 0.9212 |  | 1.23 | 0.89-1.70 | 0.2052 |  | 0.97 | 0.51-1.83 | 0.9221 |
| ***South Central*** | 0.73 | 0.47-1.15 | 0.1789 |  | 0.92 | 0.63-1.35 | 0.6763 |  | 0.76 | 0.37-1.57 | 0.4524 |
| ***London*** | 0.91 | 0.61-1.36 | 0.6442 |  | 1.01 | 0.71-1.45 | 0.9391 |  | 0.76 | 0.38-1.51 | 0.4274 |
| ***South East Coast*** | 1.13 | 0.77-1.65 | 0.5215 |  | 1.10 | 0.78-1.56 | 0.5809 |  | 1.04 | 0.55-1.97 | 0.8926 |
| ***BMI: Underweight*** | 0.99 | 0.67-1.46 | 0.9630 |  | 1.12 | 0.81-1.56 | 0.4933 |  | 0.88 | 0.49-1.59 | 0.6747 |
| ***BMI: Overweight*** | 0.71 | 0.53-0.96 | 0.0262 |  | 1.05 | 0.82-1.35 | 0.7039 |  | 1.13 | 0.73-1.75 | 0.5838 |
| ***BMI: Obese*** | 1.40 | 1.07-1.83 | 0.0133 |  | 0.93 | 0.73-1.18 | 0.5349 |  | 0.97 | 0.63-1.48 | 0.8795 |
| ***BMI: Missing*** | 0.48 | 0.07-3.39 | 0.4582 |  | 0.36 | 0.05-2.58 | 0.3103 |  | N/A | N/A | N/A |
| ***Ex-Smoker*** | 0.97 | 0.74-1.26 | 0.7998 |  | 1.11 | 0.87-1.41 | 0.3995 |  | 0.85 | 0.54-1.34 | 0.4961 |
| ***Current Smoker*** | 0.87 | 0.58-1.29 | 0.4809 |  | 0.80 | 0.54-1.18 | 0.2588 |  | 1.08 | 0.60-1.96 | 0.7909 |
| ***Smoking: Missing*** | 1.95 | 0.27-13.90 | 0.5059 |  | N/A | N/A | N/A |  | N/A | N/A | N/A |
| ***Alcohol: 1-14*** | 0.97 | 0.74-1.27 | 0.8284 |  | 1.02 | 0.80-1.30 | 0.8832 |  | 1.08 | 0.70-1.68 | 0.7238 |
| ***Alcohol: 15-42*** | 0.85 | 0.51-1.41 | 0.5291 |  | 0.70 | 0.45-1.10 | 0.1206 |  | 0.71 | 0.31-1.62 | 0.4118 |
| ***Alcohol: >42*** | 0.25 | 0.03-1.77 | 0.1649 |  | 0.70 | 0.26-1.89 | 0.4860 |  | 1.16 | 0.29-4.72 | 0.8349 |
| ***Alcohol: Missing*** | 0.90 | 0.61-1.33 | 0.6045 |  | 1.00 | 0.69-1.45 | 0.9984 |  | 1.57 | 0.90-2.75 | 0.1116 |
| ***Morbidities*** | 1.01 | 0.93-1.10 | 0.7702 |  | 1.04 | 0.96-1.12 | 0.3502 |  | 1.06 | 0.93-1.21 | 0.3663 |
| ***Prescriptions*** | 1.02 | 1.01-1.03 | 0.0001 |  | 1.02 | 1.01-1.03 | 0.0002 |  | 1.02 | 1.01-1.04 | 0.0064 |
| ***Hospitalisations*** | 1.23 | 1.00-1.51 | 0.0533 |  | 1.12 | 0.88-1.42 | 0.3600 |  | 1.40 | 1.10-1.78 | 0.0057 |
| ***Duration of diabetes (years)*** | 1.02 | 0.99-1.04 | 0.1546 |  | 1.01 | 0.99-1.03 | 0.5230 |  | 1.02 | 0.98-1.06 | 0.3515 |
| ***Complications*** | 1.65 | 1.50-1.81 | <0.0001 |  | 1.82 | 1.67-1.98 | <0.0001 |  | 1.79 | 1.54-2.09 | <0.0001 |
| ***Glucose lowering therapies*** | 1.60 | 1.41-1.83 | <0.0001 |  | 1.65 | 1.47-1.85 | <0.0001 |  | 1.60 | 1.31-1.94 | <0.0001 |
| ***Insulin prescription*** | 2.44 | 1.85-3.21 | <0.0001 |  | 2.49 | 1.75-3.56 | <0.0001 |  | 2.20 | 1.14-4.25 | 0.0194 |

Study sizes across exposures after 1:1 propensity score matching are found in Table S9, as they are the same between univariate and multivariate analyses.

Additional File 1: Table S17: Multivariate hazard ratios (with corresponding 95% CIs and p-values) for risk of sight-threatening diabetic retinopathy by each covariate across QOF exposure definitions, *among those who meet all other QOF targets*, after 1:1 propensity score matching, including the adjusted study size (n) and C-statistic (also with corresponding 95% CI).

|  | **Exposure Definition** | | | | | | | | | | |
| --- | --- | --- | --- | --- | --- | --- | --- | --- | --- | --- | --- |
|  | **Achieve HbA1c QOF Target** | | |  | **Achieve Blood Pressure QOF Target** | | |  | **Achieve Cholesterol QOF Target** | | |
|  | **Hazard Ratio** | **95% CI** | **p** |  | **Hazard Ratio** | **95% CI** | **p** |  | **Hazard Ratio** | **95% CI** | **p** |
| ***Exposure*** | 0.64 | 0.49-0.84 | 0.0015 |  | 0.88 | 0.69-1.12 | 0.2877 |  | 1.22 | 0.79-1.89 | 0.3735 |
| ***Age*** | 0.98 | 0.97-0.99 | 0.0041 |  | 0.99 | 0.98-1.00 | 0.1024 |  | 0.99 | 0.97-1.01 | 0.2312 |
| ***Sex: Female*** | 1.57 | 1.16-2.12 | 0.0031 |  | 1.39 | 1.05-1.83 | 0.0209 |  | 1.07 | 0.66-1.73 | 0.7796 |
| ***Ethnicity: Asian*** | 0.81 | 0.48-1.37 | 0.4401 |  | 0.86 | 0.51-1.45 | 0.5736 |  | 0.64 | 0.22-1.86 | 0.4089 |
| ***Ethnicity: Black*** | 1.47 | 0.71-3.03 | 0.2998 |  | 0.89 | 0.39-2.03 | 0.7905 |  | 1.67 | 0.51-5.47 | 0.3949 |
| ***Ethnicity: Mixed*** | 0.58 | 0.08-4.17 | 0.5890 |  | 1.55 | 0.57-4.21 | 0.3948 |  | N/A | N/A | N/A |
| ***Ethnicity: Other*** | N/A | N/A | N/A |  | 0.61 | 0.15-2.49 | 0.4943 |  | 0.95 | 0.13-7.02 | 0.9621 |
| ***IMD*** | 1.00 | 0.98-1.03 | 0.8836 |  | 0.98 | 0.96-1.01 | 0.1711 |  | 0.97 | 0.93-1.00 | 0.0799 |
| ***North West*** | 6.32 | 0.87-45.89 | 0.0682 |  | 1.48 | 0.59-3.72 | 0.3999 |  | 1.55 | 0.36-6.66 | 0.5539 |
| ***Yorkshire & Humber*** | 5.01 | 0.63-40.19 | 0.1289 |  | 0.49 | 0.13-1.82 | 0.2835 |  | 0.60 | 0.08-4.29 | 0.6088 |
| ***East Midlands*** | 5.38 | 0.65-44.81 | 0.1197 |  | 1.98 | 0.66-5.94 | 0.2217 |  | 1.80 | 0.30-10.94 | 0.5208 |
| ***West Midlands*** | 4.89 | 0.66-36.09 | 0.1194 |  | 1.12 | 0.43-2.91 | 0.8166 |  | 0.89 | 0.19-4.15 | 0.8866 |
| ***East of England*** | 5.73 | 0.77-42.43 | 0.0873 |  | 1.04 | 0.39-2.75 | 0.9423 |  | 0.69 | 0.14-3.52 | 0.6579 |
| ***South West*** | 4.80 | 0.65-35.29 | 0.1234 |  | 1.43 | 0.56-3.63 | 0.4499 |  | 0.85 | 0.19-3.93 | 0.8390 |
| ***South Central*** | 3.71 | 0.50-27.77 | 0.2015 |  | 1.14 | 0.44-2.98 | 0.7829 |  | 0.57 | 0.12-2.81 | 0.4939 |
| ***London*** | 4.86 | 0.66-35.80 | 0.1210 |  | 1.34 | 0.52-3.43 | 0.5428 |  | 0.76 | 0.16-3.54 | 0.7241 |
| ***South East Coast*** | 5.89 | 0.80-43.34 | 0.0818 |  | 1.38 | 0.54-3.54 | 0.5039 |  | 0.93 | 0.20-4.32 | 0.9254 |
| ***BMI: Underweight*** | N/A | N/A | N/A |  | 1.35 | 0.32-5.64 | 0.6786 |  | 0.74 | 0.10-5.78 | 0.7777 |
| ***BMI: Overweight*** | N/A | N/A | N/A |  | 1.13 | 0.28-4.63 | 0.8654 |  | 0.79 | 0.11-5.86 | 0.8159 |
| ***BMI: Obese*** | N/A | N/A | N/A |  | 0.97 | 0.24-3.99 | 0.9699 |  | 0.70 | 0.09-5.23 | 0.7275 |
| ***BMI: Missing*** | N/A | N/A | N/A |  | 0.43 | 0.04-4.74 | 0.4883 |  | N/A | N/A | N/A |
| ***Ex-Smoker*** | 0.83 | 0.62-1.13 | 0.2323 |  | 0.93 | 0.71-1.21 | 0.5850 |  | 0.66 | 0.40-1.10 | 0.1098 |
| ***Current Smoker*** | 0.66 | 0.43-1.02 | 0.0603 |  | 0.71 | 0.47-1.08 | 0.1074 |  | 0.86 | 0.45-1.65 | 0.6551 |
| ***Smoking: Missing*** | 3.20 | 0.44-23.10 | 0.2492 |  | N/A | N/A | N/A |  | N/A | N/A | N/A |
| ***Alcohol: 1-14*** | 0.85 | 0.60-1.19 | 0.3439 |  | 0.88 | 0.63-1.22 | 0.4440 |  | 1.65 | 0.80-3.37 | 0.1724 |
| ***Alcohol: 15-42*** | 0.77 | 0.43-1.37 | 0.3713 |  | 0.65 | 0.38-1.11 | 0.1163 |  | 1.12 | 0.39-3.23 | 0.8360 |
| ***Alcohol: >42*** | 0.20 | 0.03-1.47 | 0.1145 |  | 0.62 | 0.22-1.74 | 0.3631 |  | 1.71 | 0.36-8.15 | 0.5023 |
| ***Alcohol: Missing*** | 0.79 | 0.49-1.26 | 0.3202 |  | 0.90 | 0.57-1.41 | 0.6515 |  | 2.61 | 1.13-6.06 | 0.0250 |
| ***Morbidities*** | 0.80 | 0.73-0.89 | <0.0001 |  | 0.80 | 0.73-0.88 | <0.0001 |  | 0.86 | 0.74-1.01 | 0.0635 |
| ***Prescriptions*** | 1.00 | 0.99-1.02 | 0.7550 |  | 0.99 | 0.97-1.01 | 0.3445 |  | 1.00 | 0.97-1.02 | 0.7652 |
| ***Hospitalisations*** | 1.08 | 0.86-1.36 | 0.4862 |  | 1.00 | 0.77-1.30 | 0.9978 |  | 1.35 | 1.04-1.75 | 0.0249 |
| ***Duration of diabetes (years)*** | 1.01 | 0.98-1.03 | 0.5342 |  | 1.00 | 0.97-1.02 | 0.7873 |  | 1.01 | 0.97-1.05 | 0.6142 |
| ***Complications*** | 1.99 | 1.77-2.23 | <0.0001 |  | 2.13 | 1.92-2.37 | <0.0001 |  | 1.98 | 1.65-2.37 | <0.0001 |
| ***Glucose lowering therapies*** | 1.44 | 1.23-1.68 | <0.0001 |  | 1.57 | 1.34-1.84 | <0.0001 |  | 1.44 | 1.11-1.88 | 0.0067 |
| ***Insulin prescription*** | 1.45 | 1.07-1.95 | 0.0155 |  | 1.06 | 0.71-1.59 | 0.7698 |  | 0.96 | 0.46-2.02 | 0.9120 |
|  |  |  |  |  |  |  |  |  |  |  |  |
|  | **Value** | **95% CI (lower)** | **95% CI (upper)** |  | **Value** | **95% CI (lower)** | **95% CI (upper)** |  | **Value** | **95% CI (lower)** | **95% CI (upper)** |
| ***n after matching*** | 13,736 |  |  |  | 23,640 |  |  |  | 9,072 |  |  |
| ***C-statistic*** | 0.7820 | 0.7815 | 0.7824 |  | 0.8017 | 0.8014 | 0.8019 |  | 0.7859 | 0.7849 | 0.7870 |

Reference groups for categorical covariates include: white (ethnicity); North East (region); normal weight (BMI); non-smoker (smoking status); 0 units (alcohol consumption); and no insulin prescription (insulin use).

Additional File 1: Figure S1: Kaplan-Meier survival curves (and corresponding 95% CIs) for risk of diabetic retinopathy after 1:1 propensity score matching across exposure definitions.

| Exposure: HbA1c QOF Target  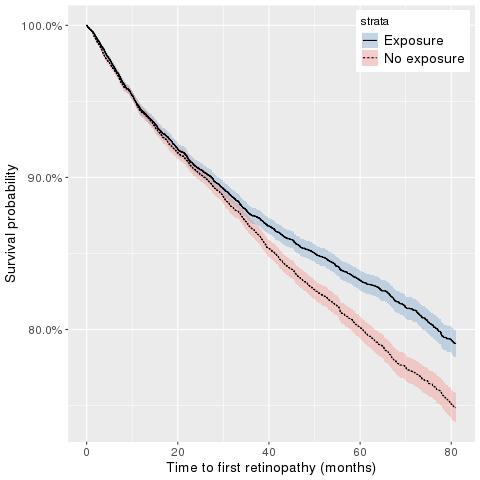 | Exposure: BP QOF Target  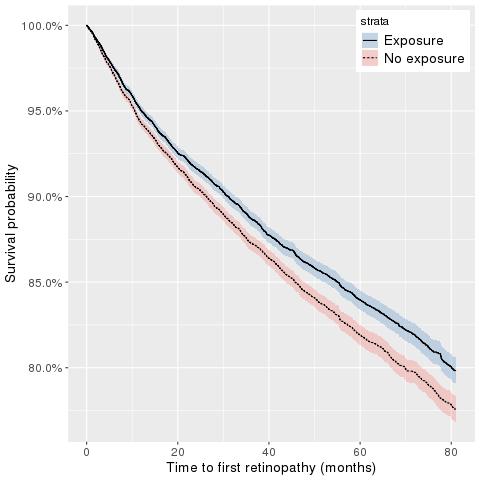 | Exposure: Cholesterol QOF Target  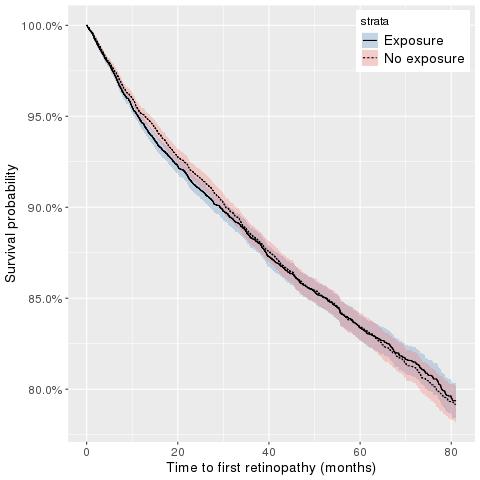 |
| --- | --- | --- |
| Exposure: 4-6 NDA Processes (vs. 0-3 NDA Processes)  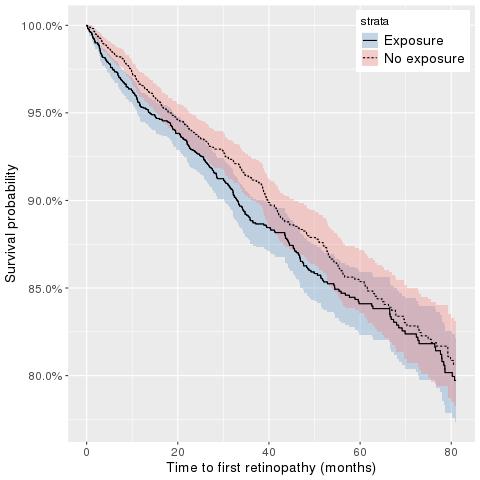 | Exposure: 7-9 NDA Processes (vs.  0-3 NDA Processes)  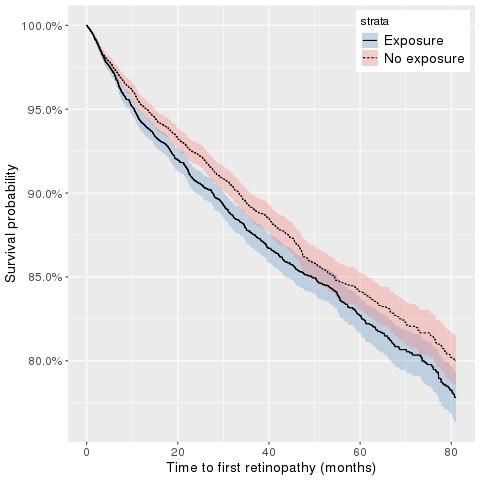 | Exposure: 7-9 NDA Processes (vs. 4-6 NDA Processes)  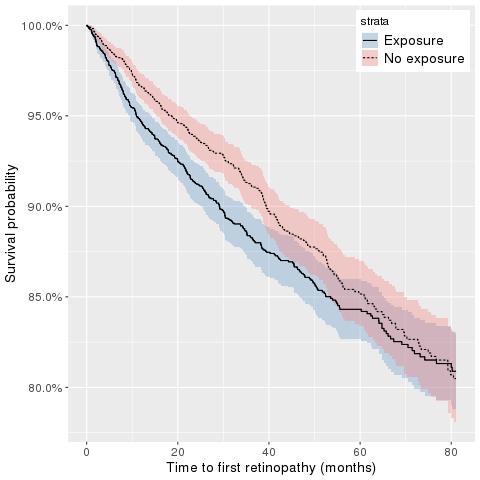 |
| Exposure: All QOF Targets  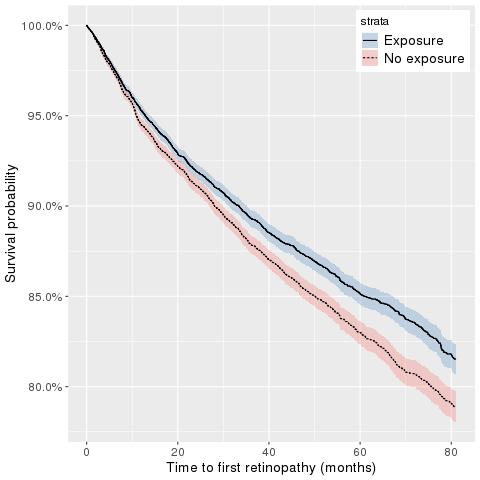 | Exposure: All NDA Processes  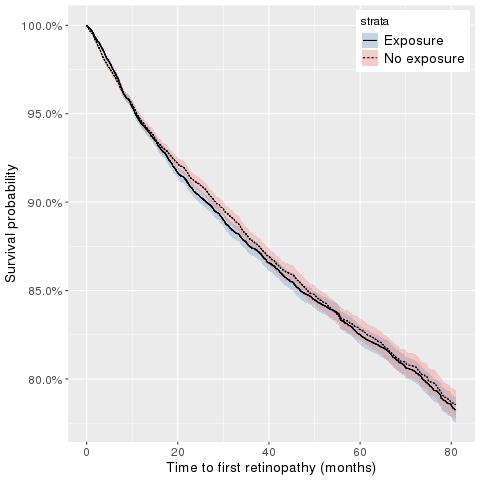 | Exposure: All QOF & NDA Targets  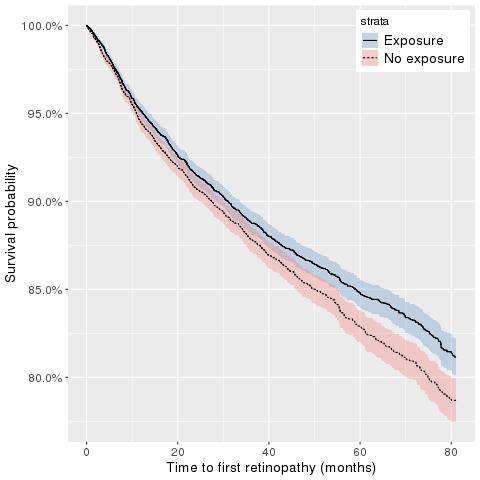 |

Additional File 1: Figure S2: Kaplan-Meier survival curves (and corresponding 95% CIs) for risk of diabetic retinopathy after 1:1 propensity score matching across QOF exposure definitions, *among those who meet all other QOF targets*.

| Exposure: HbA1c QOF Target  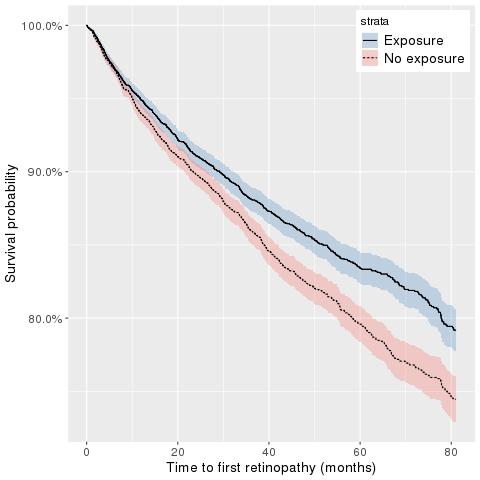 | Exposure: BP QOF Target  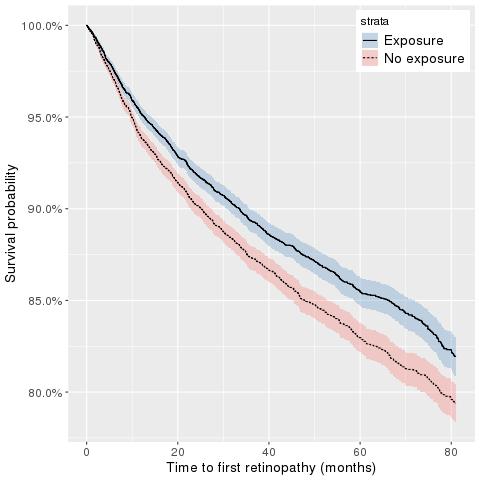 | Exposure: Cholesterol QOF Target  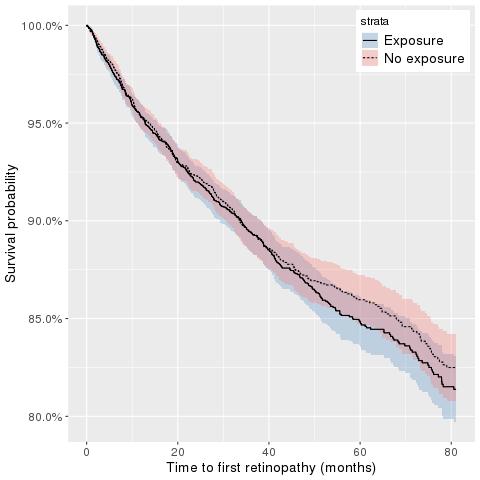 |
| --- | --- | --- |

Additional File 1: Figure S3: Kaplan-Meier survival curves (and corresponding 95% CIs) for risk of sight-threatening diabetic retinopathy after 1:1 propensity score matching across exposure definitions.

| Exposure: HbA1c QOF Target  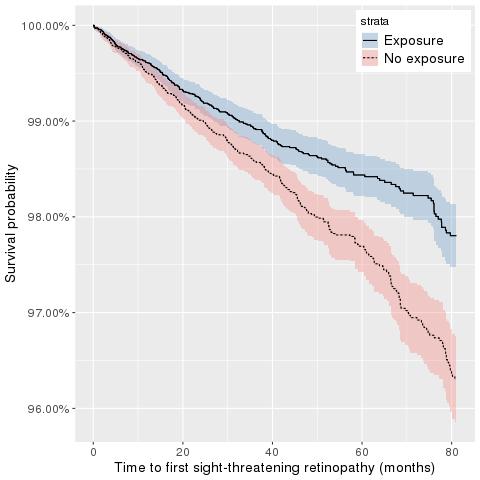 | Exposure: BP QOF Target  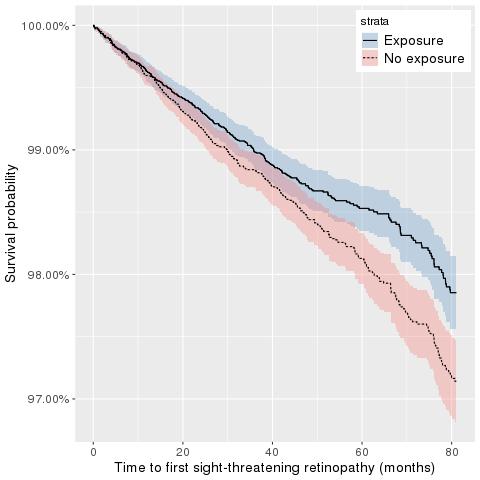 | Exposure: Cholesterol QOF Target  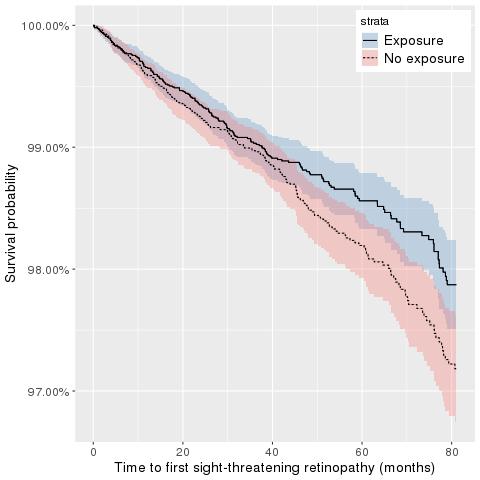 |
| --- | --- | --- |
| Exposure: 4-6 NDA Processes (vs. 0-3 NDA Processes)  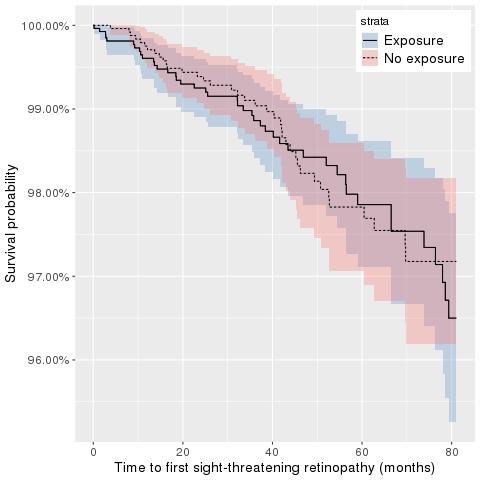 | Exposure: 7-9 NDA Processes (vs.  0-3 NDA Processes)  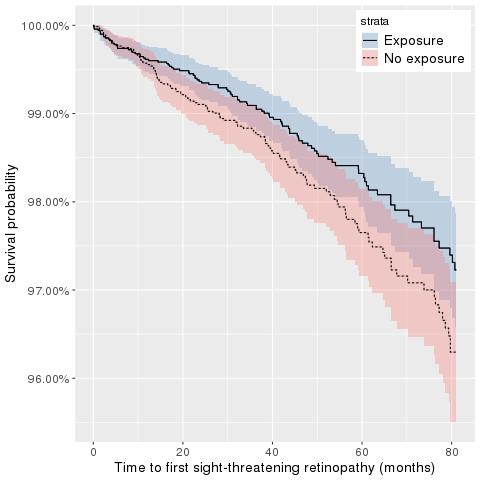 | Exposure: 7-9 NDA Processes (vs. 4-6 NDA Processes)  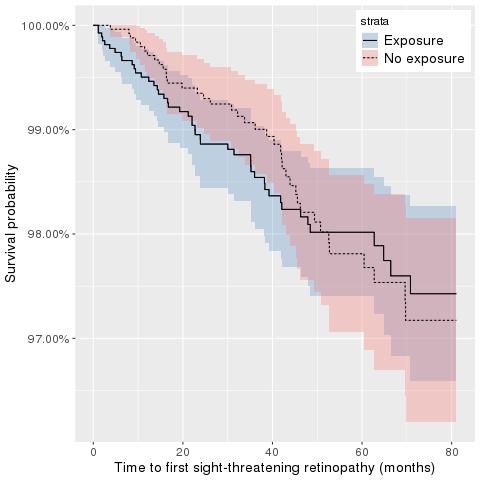 |
| Exposure: All QOF Targets  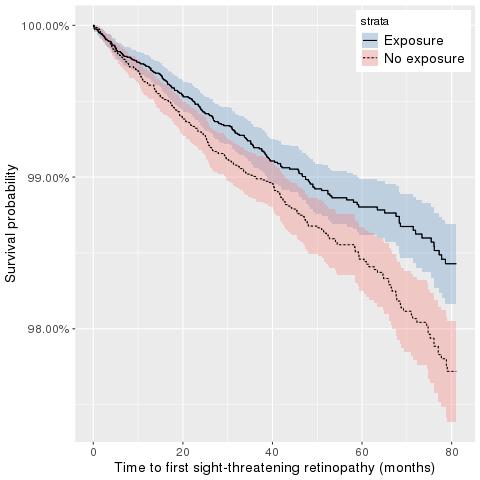 | Exposure: All NDA Processes  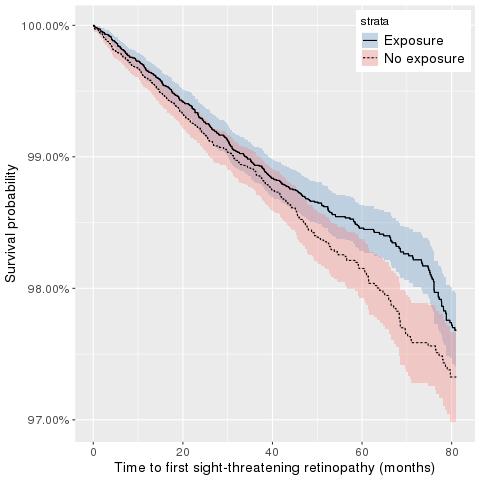 | Exposure: All QOF & NDA Targets  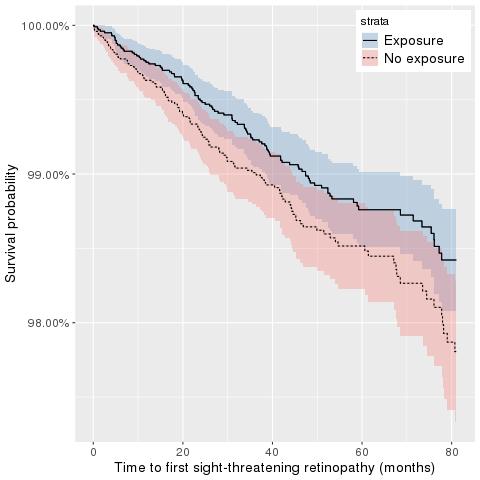 |

Additional File 1: Figure S4: Kaplan-Meier survival curves (and corresponding 95% CIs) for risk of sight-threatening diabetic retinopathy after 1:1 propensity score matching across QOF exposure definitions, *among those who meet all other QOF targets*.

| Exposure: HbA1c QOF Target  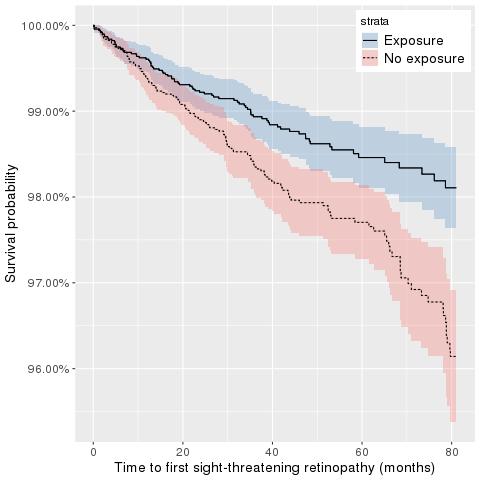 | Exposure: BP QOF Target  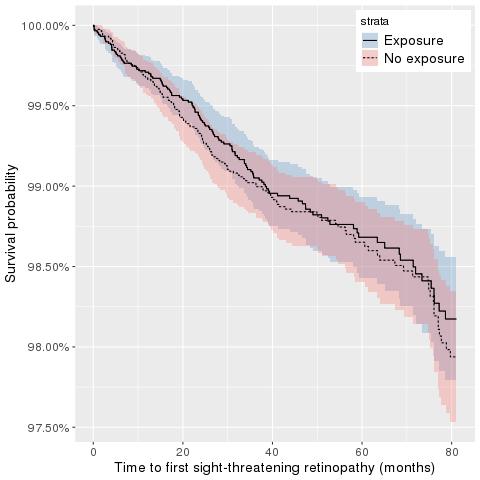 | Exposure: Cholesterol QOF Target  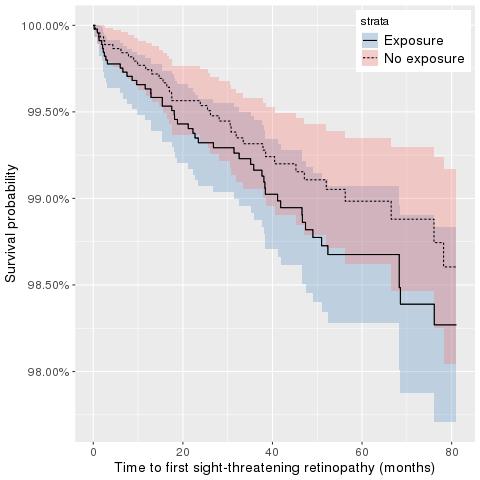 |
| --- | --- | --- |
